# Phase transitions perception in nonreciprocal mechanical metamaterials through electromagnetic resonance

**DOI:** 10.1126/sciadv.ady1211

**Published:** 2025-09-12

**Authors:** Yun Deng, Xiaoyu Zhao, Zhixin Huang, Ying Li

**Affiliations:** ^1^ School of Naval Architecture, Ocean and Energy Power Engineering, Wuhan University of Technology, Wuhan 430063, Hubei, China.; ^2^ State Key Laboratory of Explosion Science and Technology, Beijing Institute of Technology, Beijing 100081, China.; ^3^ Beijing Key Laboratory of Lightweight Multi-functional Composite Materials and Structures, Institute of Advanced Structure Technology, Beijing Institute of Technology, Beijing 100081, China.

## Abstract

Phase transitions of metamaterials are critical in advancing energy conversion efficiency and controlling mechanical performance. However, the design method and localized perception of phase transitions remain challenging. Inspired by the passive coupling mechanisms in ostrich locomotion, this work proposes nonreciprocal metamaterials that can perceive real-time phase transition. These architectures enable the topological solitons to propagate unidirectionally and overcome dispersive and dissipative effects through bistable-to-monostable state switching between adjacent units. The integration of electromagnetic resonators within the metamaterial units enables real-time detection of dynamic phase transitions, as soliton propagation or external loads induce resonance frequency shifts between distinct stable states. By arraying these mechanoreceptive units and the combination of the machine learning, it can encode information and compute programmatically. Furthermore, the mechanoreceptors hold promising applications in robotics. This work provides an approach for integrating phase transition perception and nonlinear wave manipulation and offers insights into dynamic material intelligence and energy management systems.

## Teaser

Electromagnetic resonance enables real-time perception of phase transitions in mechanical metamaterial.

## INTRODUCTION

Phase transitions play a critical role in enhancing energy conversion and regulating mechanical performance, exemplified by the martensitic-austenitic transition in ferroelectric materials (*1*–*3*) and the domino-like folds in twisted monolayer-multilayer graphene (*4*, *5*). Specifically, small-angle twisted monolayer-multilayer graphene exhibits two metastable reconstructed states with distinct stacking orders and strain soliton structures. The localized phase transitions can spontaneously propagate over long distances through coupled deformation effects within the soliton networks. Such transformations (*6*, *7*) enable precise control over energy conversion, dissipation, and system damage prevention. Phase transitions hold promise for energy management devices, including sensing (*8*), dynamic shape morphing (*9*), and robotic manipulation (*10*, *11*), yet their design method and localized perception remain challenging.

Mechanical metamaterials offer a natural platform to design phase transition materials. In particular, topological solitons (*12*, *13*) in dissipative systems exhibit resilience against damping, enabling robust energy and information transport. These advantages are taken by mechanical configurations with snap-through diffusive kinks (*14*, *15*) and soft elastic beams (*16*, *17*), shells (*18*, *19*). The propagation of these diffusive kinks is governed by a reaction-diffusion mechanism, where the local energy landscape is modulated by the viscoelastic properties and geometric imperfections of the kirigami. This viscoelastic, noninertial propagation inherently results in slow kink movement, which may limit the efficiency of dynamic applications requiring rapid actuation. On the contrary, the soft beams use the inertial propagation to release stored elastic energy in the bistable elements. The topological solitons propagate along a multiwalled energy landscape created by an array of bistable elements, each of which switches between their two stable states to propagate the transition front. Similarly, the strain energy landscape of universally bistable shells can be tuned by the geometric parameters. These systems use the inertia to release the elastic energy strain and the extra amount of supplied energy to initiate the solitons. The bistable properties of these structures rely on the fixed boundary conditions or rigid chambers. A natural question is how to design the inertial propagation bistable units to without relying on fixed boundaries, thereby expanding the application scenarios of transition waves.

To enhance the intelligence of bistable units, thermal or electromagnetic stimuli (*20*–*22*) are used to encode mechanical properties such as shapes (*23*), adaptive impact resistance (*24*), customizable constitutive responses (*25*, *26*), curvatures (*27*), and stiffness (*28*–*30*), offering greater advantages when combined with approaches beyond the range of elasticity. Specifically, embedding magnets (*7*, *31*) or magnetic particles (*32*) within elastic structures enables reversible phase transitions and programmable transition waves. In this context, other approaches have integrated stimuli-responsive smart materials, such as temperature-responsive kirigami (*33*, *34*), to trigger the propagation of transition waves and control phase transitions in multistable structures. Classical theoretical models (*35*–*37*) provide a well-established framework for predicting phase transitions in reconfigurable mechanical metamaterials. However, rare studies (*38*–*40*) have offered notable insights into the integration of nonlinear waves control and phase transition perception. Nature provides many examples of real-time motion sensing. For instance, human skin (*41*) contains numerous mechanoreceptors that transmit information and enable perception. Similarly, running animals (*42*) sense interaction forces at their feet to maintain stability on uneven terrain. Bioinspired artificial mechanoreceptors (*43*–*45*) offer an efficient method for real-time monitoring. Artificial mechanoreceptors that convert mechanical motions into electrical signals have emerged as versatile tools, finding widespread applications in bioengineering (*46*, *47*), medical diagnosis (*48*, *49*), and ocean exploration (*50*, *51*). Compared with other mechanoreceptors, electromagnetic resonance–based artificial receptors offer the advantages of high sensitivity (*52*, *53*), strong environmental adaptability (*54*, *55*), and integrability (*56*, *57*). The integration of electromagnetic resonance–based artificial mechanoreceptors with bistable mechanical metamaterials represents a promising route for enabling real-time sensing and control of nonlinear wave dynamics and phase transitions.

To address the challenge of design method and enabling localized phase transition perception, we designed and fabricated an architectural structure composed of soft highly dissipative materials and hard minimally dissipative materials in this study. These nonreciprocal mechanical metamaterials can effectively overcome both dispersive and dissipative effects and enable the propagation of mechanical signals over arbitrary distance by leveraging geometric coupling between adjacent units to eliminate the maximum structural energy barrier. Analytical, numerical, and experimental results indicate that the mechanical properties of the next unit are influenced by the previous unit based on a contact-induced topological transformation. On this basis, the phase transitions and unidirectional propagation of nonreciprocal topological solitons are achieved without external stimulus. The wave velocity could be customized through precise design of the bistable unit. Furthermore, to attain real-time perception of phase transitions in metamaterials, we harness electromagnetic resonance and reconfigurable characteristic of metamaterials to construct an artificial mechanoreceptor. These mechanoreceptors are used to design sensor arrays capable of pixel encoded information processing and self-sensing robots capable of directed motioning, which further highlights the potential and adaptability of design strategy.

## RESULTS

### Design strategy of the nonreciprocal mechanical metamaterials

**Fig. 1.**
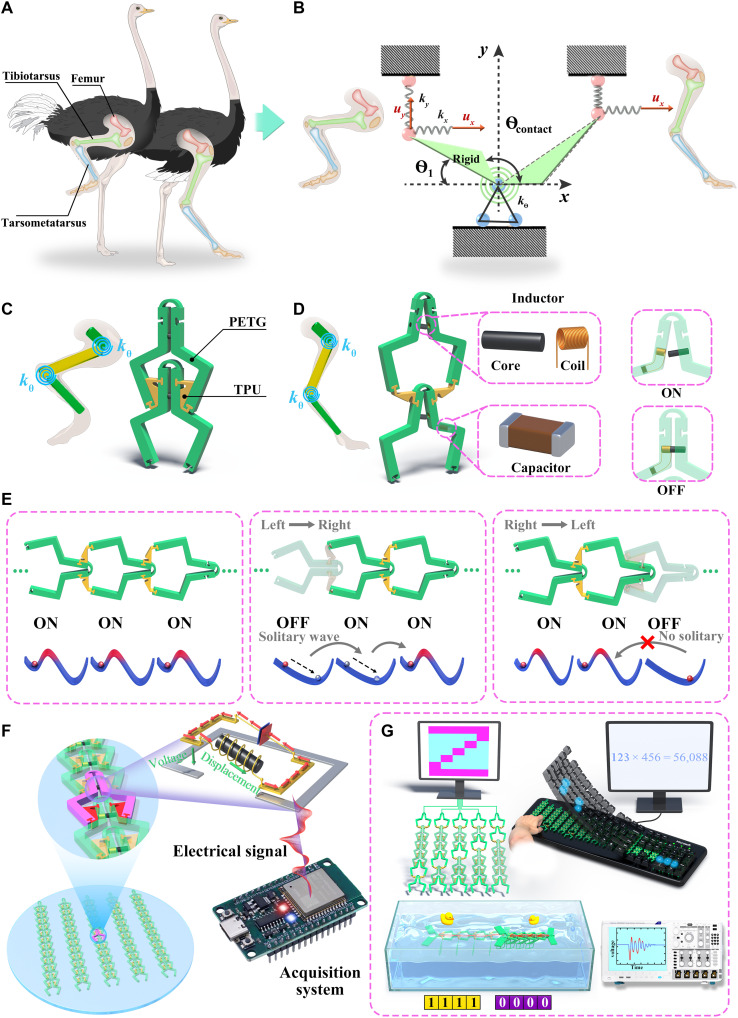
Design strategy and overview of the nonreciprocal mechanical metamaterials. (**A**) In the walking ostriches, the legs are toes extend in swing and digital-extended upon knee extension in preparation for stance. (**B**) A simplified multibody system constrained by hinges with a rotational spring *k*_θ_ at bottom and two grounded springs *k_x_* and *k_y_* at the top. (**C**) Nonreciprocal mechanical metamaterials evolved from this system. (**D**) Artificial mechanoreceptors integrating electromagnetic components to achieve the real-time phase transition perception. (**E**) Dynamical responses of the metamaterials: (i) from left to right and (ii) from right to left. (**F**) Scalable arrays for phase transition perception and advanced information processing. (**G**) A self-sensing soft robot based on the nonlinear wave and electromagnetic resonance.

In the natural world, insects like *Drosophila* (*58*) have not evolved mechanically self-stable structures to passively support their body weight and rely on continuous muscle tension to maintain a standing posture. In contrast, vertebrates such as ostriches (*59*) have leg skeletons with natural mechanical support points. A knee-locking mechanism enables stable standing in ostriches with minimal muscle effort. Structurally, the ostrich leg mainly consists of the femur, tibiotarsus, and tarsometatarsus, connected by strong joints. Leg flexion occurs through rotation of this bone around a proximal synovial. As shown in Fig. 1A, a multiarticular spring network guides leg trajectory and provides rapid transition between stance and swing phases. The ligaments provide tension during weight support. Contact among the femur, tibiotarsus, and tarsometatarsus ensures that skeletal displacements remain within a rational range through a mechanism reminiscent of self-engaging clutch (*59*), thereby maintaining postural stability. Inspired by this phenomenon, we propose a simplified multibody system to model these rapid phase transition mechanisms in locomotion system. The system constrains by hinges with a rotational spring *k*_θ_ at bottom side and two grounded springs *k_x_* and *k_y_* in the upper end (see Fig. 1B). Upon applying a horizontal displacement to the system, the rigid body undergoes rotational and parallel motion. If the rotational and grounded springs exhibit sufficiently high stiffness, in combination with the implementation of contact constraints, then the system can finely regulate its mechanical response, transitioning seamlessly between open and closed states.

**Fig. 2.**
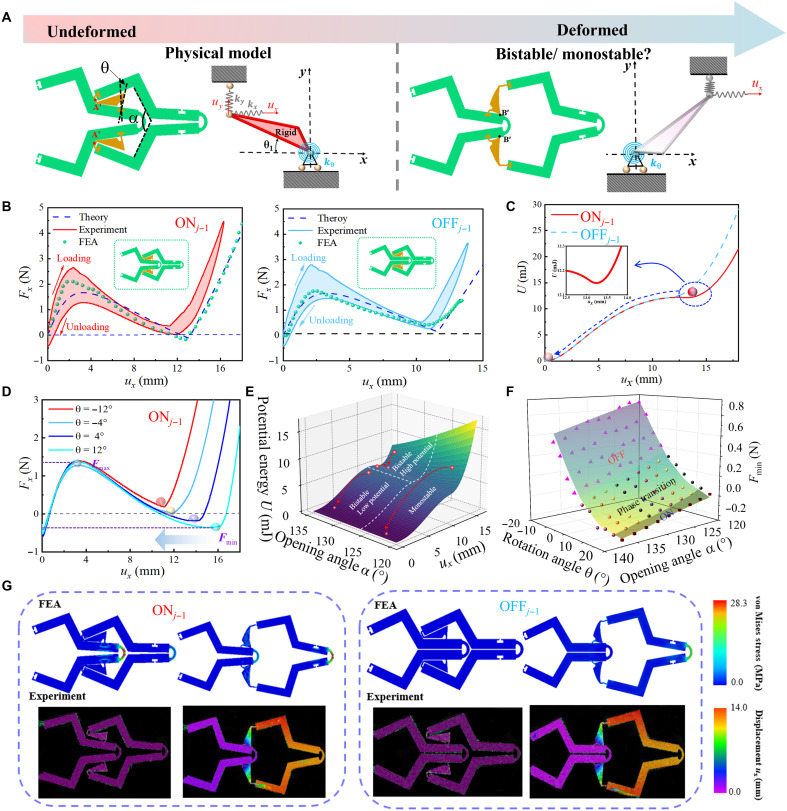
Experimental, numerical, and theoretical characterization of the multibody architecture. (**A**) Simplified mechanical model of metamaterials similar to the action of a self-engaging clutch. (**B**) The mechanical behavior of the *j*th unit cell during opening or closing of the (*j* − 1)th unit cell. (**C**) The energy barrier of the *j*th unit cell during opening and closing of the (*j* − 1)th unit cell. (**D**) The mechanical properties of the *j*th unit cell under different the opening angle θ (−12°, −4°, 4°, and 12°) at the same rotation angle α = 135°. (**E**) Structural monostable and bistable activation pathways. (**F**) The influence of the opening angle θ and rotation angle α on the phase transition region. (**G**) The von Mises stress and deformation mode of the *j*th unit cell during opening and closing of the (*j* − 1)th unit cell.

Taking the mechanical system of the ostrich leg as a model, we abstract it as a simplified architecture (Fig. 1C). The femur and tarsometatarsus correspond to the polyethylene terephthalate glycol (PETG) segments of *j*th and (*j* + 1)th units, respectively, while the tibia is represented by the thermoplastic polyurethane (TPU) segment. The two hinges within the TPU component mimic ligaments located at the joints, and the contact interface between the TPU and PETG segments corresponds to the pivot point between the femur and tibia. When the system is in a high-energy potential state, the TPU connector contacts with the upper part of the framework (the point B′), preventing further movement (see Fig. 2A and fig. S5). In contrast, as the system is at a low-energy potential state, the TPU connector contacts the lower part of the framework (the point A′), restricting movement. Between these contact points, the TPU connector is able to move, altering the energy state of the structure and driving motion, similar to the action of a self-engaging clutch.

Based on this principle, two commercially 3D printing polymers are used to construct the mechanical metamaterials, because the single-material structures are improper to simulate bones and ligaments with both rigidity and flexibility as shown in Fig. 1C. Hinges fabricated solely from PETG tend to exhibit significant plastic strain even fracture during rotation. Conversely, those made entirely of TPU fail to provide sufficient stiffness during rigid body contact, compromising stability and preventing structural phase transitions. Thus, we use PETG with higher rigidity as the unit framework and complement by TPU as the soft hinges. This design ensures that PETG provides the necessary stiffness for TPU soft hinge to enable stable states through contact-induced topological transformation during the rotational process. To achieve the real-time phase transition perception, we construct an artificial mechanoreceptor by integrating electromagnetic resonance with nonreciprocal metamaterials (Fig. 1D). The metamaterial exhibits distinctly different resonance frequencies when transforming between the open and closed states, serving as the primary feature for identifying phase transitions.

The energy barrier in metamaterial depends on the mechanical properties of the preceding unit (Fig. 1E). When the unit cell forms a long chain, the (*j −* 1)th unit crosses over its highest energy barrier from left to right. This causes the maximum energy barrier in the *j*th unit to disappear. In contrast, as the (*j* + 1)th unit overcomes its energy barrier, the *j*th and (*j* − 1)th units remain mechanically unchanged. This phenomenon is defined as intrinsic nonreciprocity. Furthermore, we extend the design of artificial mechanoreceptors into scalable arrays (Fig. 1F) for phase transition sensing and advanced information processing. By integrating electromagnetic resonance and machine learning with mechanically coupled metamaterials, the array emulates key functions of commercial keyboards, including information writing, erasure, and computation with high accuracy. On this basis, we harness topological soliton to power the aquatic robot and electromagnetic resonance to perceive its motion state, thereby realizing a self-sensing robot driven by nonlinear waves (Fig. 1G).

### Kinematics theory for multibody architecture

As depicted in Fig. 2A, the multibody system constrained by hinges, featuring a rotational spring *k*_θ_ and two grounded springs *k_x_* and *k_y_*. The kinematic framework of multibody system is derived in detail on the basis of the energy method (see equations S6 to S12 in Supplementary Text S2), characterizing the influence of springs (*k_x_*, *k_y_*, and *k*_θ_) and contact interaction $\theta_{\text{contact}}$ on its performance. Results show that larger contact angle favors the system in achieving the stable state. After unveiling the activation mechanism of contact interaction, we use the multibody architecture to develop the mechanical metamaterials capable of switching monostable and bistable. The representative mechanical responses of unit cell (see Fig. 2A and fig. S2) are governed by the geometric parameter, i.e., the opening angle α and rotation angle θ. On this basis of extensive theoretical calculations (see figs. S3, S4, and S6), the stiffness of three springs is designed: *k_x_* = 1.278 N/mm, *k_y_* = 0.281 N/mm, and *k*_θ_ = 3.449 (N·mm)/rad.

Figure 2B illustrates the mechanical responses exhibited by the *j*th unit, when controlling the motion of (*j* − 1)th unit via adjusting the opening angle α, which can take either open or closed states. The unloading force-displacement curves reflect spontaneously phase transition. Specifically, the *j*th unit exhibits a monostable and bistable mechanical response, while the (*j* − 1)th unit is respectively closed and open. Notably, the loading and unloading curves do not fully overlap, primarily due to the viscous hysteresis of TPU during the experiment, as evidenced by tensile-relaxation tests of the parent materials (fig. S1). The digital image correlation and finite element analysis (FEA) (Fig. 2G and fig. S8D) show that the maximum strain of TPU and PETG in metamaterials is 0.387 and 0.028, respectively. The PETG deformed elastically during the tests, because the strain of structure is smaller than the elastic limit. In addition, we have conducted comparison tests for the metamaterials with PETG hinges and TPU ligaments as shown in fig. S8A. The small difference is observed for the loading and unloading curves with PETG hinges, while obvious differences are occurred for the TPU-based designs. It should be mentioned that, although the hysteresis effect exists, the loading and unloading curves are approaching stability after the first cycle (fig. S8, A and B), which means that the difference between the curves in the previous and next cycle is minimal. This feature can ensure the repeatability of the performance.

The energy landscape of the metamaterial during the opening and closing states is further analyzed. As plotted in Fig. 2C, the *j*th cell exhibits two energy potential wells during opening stage, whereas it only displays a single potential well in the closed state. This indicates that the previous (*j* − 1) unit converts from the opening to closing stages can eliminate the peak of the local energy in the current *j* cell, thereby releasing the stores strain energy within the structure. To quantify the effect of the contact (controlled by the rotation angle θ, where counterclockwise rotation is defined as positive) on the deformation of the metamaterial, we experimentally and theoretically observe that the *j*th unit exhibits different mechanical responses (unloading curves) at various rotational angles (−12°, −4°, 4°, and 12°) when the (*j* − 1)th unit is open (see Fig. 2D and fig. S10). At excessively large negative rotational angles, the soft hinges come into premature contact with the PETG, causing instability. In contrast, overly large positive rotational angles result in an overly stable structure, preventing the occurrence of spontaneous phase transitions.

Achieving spontaneous phase transitions in metamaterials, we inspect the energy landscape of the architecture during activation in Fig. 2E. The opening angle α of the previous (*j* − 1) unit exceeds 126°, the current *j* unit becomes trapped in a local minimum potential landscape, inducing a bistable state within the structure. This requires an external input to cross the energy barrier to convert from the high-energy barrier potential to the low-energy barrier potential. Conversely, when the opening angle is decreased to below 126°, the metamaterial rapidly transitions from the bistable to monostable state, thereby triggering structural phase transition. Additionally, the rotation angle θ modulates contact stiffness to influence the spontaneous phase transitions. Figure 2F presents the impact of different rotation and opening angle on the structural phase transition. The phase transition is primarily influenced by the rotational angle of the hinge. For a fixed opening angle, the phase transition (ON and OFF stages) is triggered when the rotation angle within the spontaneous range of (−10°, 10°). At rotation angle of the metamaterial less than −10°, the structure resides in OFF phase, where it is unable to maintain a stable phase during opening, regardless of other conditions. On the contrary, when the rotational angle exceeds 10°, the structure enters ON phase, indicating the stable configuration. Owing to this phase transition, the metamaterial exhibits nonreciprocal mechanical responses in its dynamical behavior.

### Dynamical model of nonreciprocal metamaterials

**Fig. 3.**
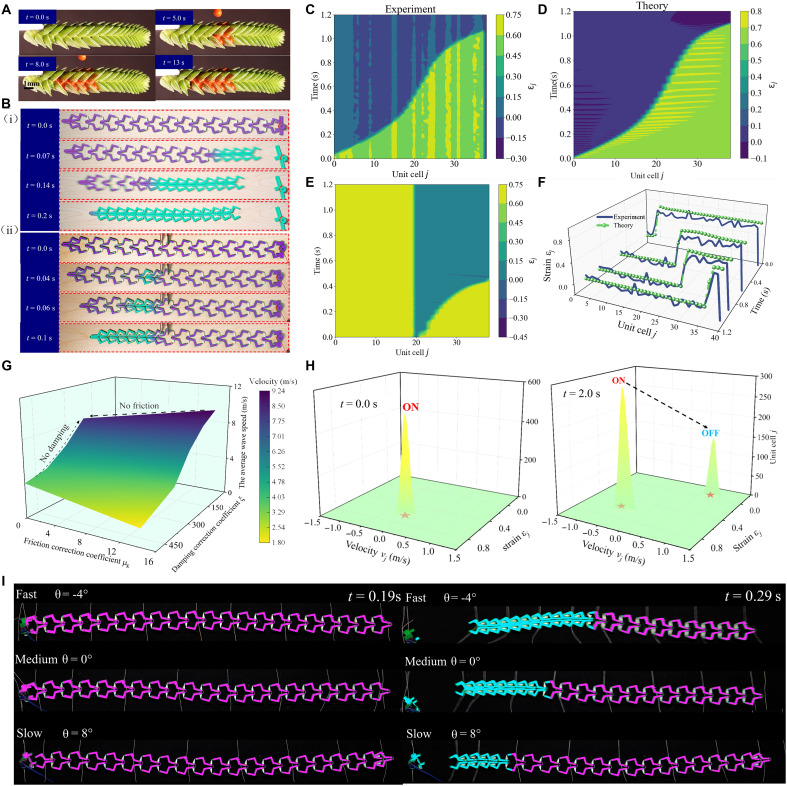
Dynamic model of nonreciprocal mechanical metamaterials. (**A**) Optical image showing ethanol (stained red) being guided to the left by the *C. muscosa*, but not to the right. (**B**) Nonreciprocal mechanical metamaterials consisting of 14 identical unit cells exhibits nonreciprocity in dynamic model: (i) from right to left and (ii) from left to right. (**C**) Theoretical model and (**D**) experimental study on the evolution of the strain of each bistable unit in homogeneous transmission. (**E**) The strain evolution of each bistable unit under intermediate activation, reflecting the nonreciprocal characteristics toward waves of structure. (**F**) Comparison of the phase transformation regions between theory and experiment at different times. (**G**) The effect of structural damping and ground friction on the transition wave speed. (**H**) Evolution of the strain and velocity of 500-unit cells during the propagation of the transition wave (*t* = 0 s and 2 s). (**I**) The three different wave velocities: fast, medium, and slow in metamaterials.

Eliminating the highest energy barrier in bistable systems can trigger structural phase transitions, thereby spontaneously generating transition waves. This phenomenon is analogous to the directional liquid flow observed in *Crassula muscosa* (*60*). It is achieved through asymmetric concave leaf structures with varying angles, enabling the unidirectional transport of specific liquids along a fixed direction (see Fig. 3A and movie S2). Inspired by this biological mechanism, the nonreciprocal mechanical metamaterials are designed to support the unidirectional propagation of topological solitons, based on the theoretical multibody kinematics framework. We first created a one-dimensional (1D) periodic lattice chain of mechanical metamaterials consisting of 15-unit cells [Fig. 3B (i)]. A sufficient large displacement applied to the chain can cause the initial element to transition states, producing a nonlinear transition wave from the point of initiation. The propagation of transition wave in the lattice is observed without any external physical field (see movie S3). Experimental and finite element results (in fig. S12) confirm the nonreciprocity wave modulation of the structure. Distinct nonlinear wave dynamics are observed when waves propagate from right to left versus left to right. Transition wave initiates from the right end of the chain propagate exclusively to the left [see Fig. 3B (ii) and movie S4], without reverse propagation. This behavior is similar to the directional liquid flow guided to the left as illustrated in Fig. 3A.

To experimentally characterize the propagation mechanism of such nonlinear waves, we used a camera to track each bistable element’s location along the chain comprising 40 units as the function of time (fig. S13; see movie S5). All units are initially in their higher-energy stable configuration at the ON phase (noting that the last unit, lacking coupling effect of adjacent cell, remains in the OFF phase). Then, at time *t* = 0 s, the first unit (ON phase) is applied a sufficient displacement into the OFF phase. We find that the initial configuration is unstable and the units in the higher-energy minimum (phase ON) sequentially transition to the lower-energy deformed stable state (phase OFF), producing a nonlinear transition wave that propagates toward the right with nonconstant velocity (see Fig. 3C). Note that the method of generating wave differs from those observed in 1D mechanical metamaterials (*16*, *32*) comprising an array of coupled bistable units with a constant velocity. These adjacent units lack strongly interaction coupling. Our chain achieves soliton transmission by eliminating the energy barrier of current unit through the action of the preceding unit.

On this basis, we further derive the governing equations using a generalized Lagrangian formulation and solve them numerically to comprehensively characterize the transition wave propagation. To this end, we focus on the *j*th unit cell and use the horizontal displacement *u_x_* as the mechanical property transmission variables between adjacent units. The dynamical equation comprises four components: elastic and damping forces from the springs on both sides, inertial force of unit cell, and frictional force from the ground. The detailed derivation is described in Supplementary Text S3 (equations S13 to S23). The results (Fig. 3, C, D, and F) show that the theoretical model aligns well with the experimental results. Theoretical phase transition strain in metamaterials slightly exceeds the experimental observations. This discrepancy primarily arises from the errors between the theoretical and experimental solutions in the kinematic model of the nonreciprocal metamaterials. In the initial stage (approximately the front unit cell 11), the wavefront propagates at a constant transition wave velocity of *c*_1_ = 2.57 m/s (see fig. S14B), reflecting the faster propagation phase, which is less affected by structural damping or friction. At the subsequent segment and third region, the wave propagation slows down to *c*_2_ = 1.39 m/s and *c*_3_ = 0.84 m/s. However, the wave reaccelerates to the same velocity as *c*_1_ at the end of chain (see movie S5). The variation in propagation speed among different units in the system is associated with boundary effects and interunit variations. During the transition from a high to low energy barrier state, each unit releases energy. This released energy generates a dragging force that can induce motion in neighboring units, regardless of whether they are in high or low energy states. As more units are dragged into motion, the sum of dynamic friction acting on the system increases accordingly. According to the dynamitic equations (equations S22 and S23), increased friction leads to a reduction in both acceleration and velocity. As the systems evolve over time, the dragging force at right end of the chain becomes smaller than at the left end, and right end of the chain begins to contract. Consequently, the transition wave accelerates, reaching a velocity comparable to the initial propagation speed *c*_1_. Notably, theoretical model also demonstrates (see Fig. 3E) the nonreciprocal property of metamaterial. When the displacement excitation is applied to the 19th unit cell, the transition wave propagates to the right, reaching the final unit. Meanwhile, all units on the left remain at the high-energy barrier equilibrium. Figure 3F shows the strain evolution across 40-unit cells over time, with wavefronts propagating sequentially.

After characterizing the nonlinear dynamical behavior of metamaterials, we quantitatively investigate how structural damping and friction affect the nonlinear wave mechanism. The average transition wave speed is used to describe the interaction between damping ξ and friction μ*_k_* correction coefficient (see Fig. 3G). When friction is removed from the system (μ*_k_* = 0), transition wave propagates through the metamaterials at a constant speed of *v* = 3.87 m/s (see fig. S14A). This result aligns with most findings (*12*, *18*) in the literature, indicating that the metamaterial achieves dynamic equilibrium between dispersive and nonlinear effects. Consequently, the strain energy is converted into the kinetic energy associated with the motion of individual unit cells in the system. The metamaterials overcome both dispersive and dissipative effects and enable the propagation of a mechanical signal over arbitrary distances without distortion. Transition waves do not propagate at a constant speed when the system decreases the damping function but retains the friction term. Instead, their average wave speed decreases as the friction correction coefficient increases. The strain energy transfers to the kinetic energy and internal energy of the unit cells. To better understand the phase distribution, we simulate the chain’s dynamical response consisting of 500 units. Figure 3H illustrates that the phase evolution of strain ε_*j*_ and velocity *v*_*j*_ (*v*_*j*_ denoting the velocity of the *j*th unit cell) at two different time steps (*t* = 0.0 s and *t* = 2.0 s). Five hundred–unit cells are in the “ON” state, characterized by a strain ε_*j*_ ≈ 0.8 and velocity *v*_*j*_ = 0 m/s at initial stages. Then, a clear transition is observed as certain unit cells switch to the “OFF” states. They transition to the lower-energy stable configuration at ε_*j*_ ≈ 0.014 and acquire a velocity of *v*_*j*_ ≈ 1.12 m/s. Results demonstrate that transition waves can be generated by eliminating the energy barrier peaks of adjacent structures, where individual units reside in separate phase transformation regions during motion.

Using these dynamical characteristics of the theoretical model, we experimentally demonstrate that the transition wave velocity could be customized through tuning geometric parameters of the bistable unit (the rotation angle θ and ligament thickness of *t*_PETG_). Friction is sensitive to the external environment and is unsuitable to prove that the wave velocity could be controlled by the precise design of metamaterials. We adopt a suspended setup to construct a nonfriction experimental environment, which trigger the transition wave to measure wave velocity. The units (see fig. S15) with three types of geometrical parameters are used to demonstrate wave propagation at distinct velocities within the system. Figure 3I and movie S6 show three transition wave velocities (fast, medium, and slow) that pass through the metamaterial, gradually transforming unit cells from the open to closed equilibrium state. The transition wave velocity is mainly be influenced by the elastic force and the damping force. Note that the elastic force arises from the hyperelastic deformation of TPU and linear elastic deformation of PETG. It is primarily governed by the ligament thickness of the PETG components, whereas the damping force originates from the viscoelastic dissipation of TPU and is mainly determined by structural design parameters of the TPU ligaments. It can be observed that the unit (the thickness *t*_PETG_ = 1.0 mm of PETG and rotation angle θ = −4°) produces the highest constant wave velocity *c* = 4.18 m/s (see fig. S17A) in contrast to other situations (*c* = 3.02 m/s in fig. S17B and *c* = 1.63 m/s in fig. S17C). A thicker PETG ligament [the rotation angle θ (−4°,0°,8°) controls the bistable state of units] generates a larger elastic force, which requires a correspondingly greater damping force to achieve dynamic balance. This interplay results in a higher transition wave velocity. These experimental results also demonstrate that the friction is the main reason to nonconstant wave velocity of metamaterials (see Fig. 3C).

### Phase transition perception via electromagnetic resonance

**Fig. 4.**
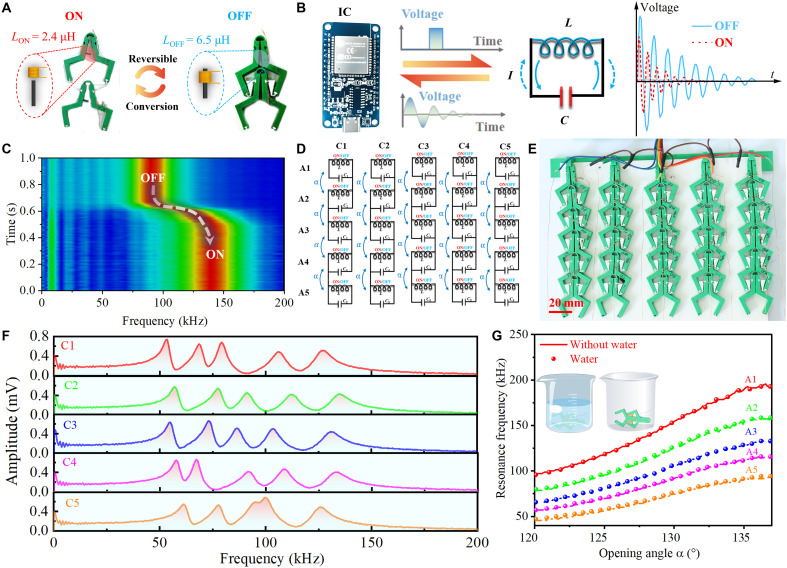
The artificial mechanoreceptor of nonreciprocal metamaterial based on electromagnetic resonance. (**A**) The bistable unit cell exhibits two different inductance characteristics during the ON and OFF states. (**B**) The schematic diagram of a resonant circuit shows two different resonant frequencies in this bistable unit cell. (**C**) The dynamic frequency response of the system over time as it transitions between the ON and OFF states. (**D**) The equivalent circuit diagram of the mechanoreceptor array: Every single chain couples the resonance characteristics of adjacent bistable units through the opening angle α. (**E**) Photograph of 5 pixel–by–5 pixel planner mechanoreceptor array. (**F**) The frequency-amplitude response of every bistable chain (from left C1 to right C5). (**G**) The effect of unit cell position (from top A1 to bottom A5) within the chain and the influence of extreme environments (with and without water) on the relationship between resonance frequency and opening angle.

After unveiling the dynamical performance of the mechanical metamaterials, the multibody architecture is further used to develop an artificial mechanoreceptor based on electromagnetic resonance, as shown in Fig. 4A. The electromagnetic resonance can converse the mechanical responses into electronic signals. The mechanoreceptor’s inductance switches reversibly between *L*_ON_ = 2.4 μH and *L*_OFF_ = 6.5 μH through the bistable response of units. Figure 4B depicts the operational principle (the time resolution of mechanoreceptors is ~660 μs; see fig. S19). The mechanical deformation induces the inductor-capacitor resonant shifts from the ON to OFF states corresponding to higher and lower frequencies, respectively. As the cell open, the magnetic core follows the deformation and withdraws from the coil, which decreases inductance and increases the resonance frequency. The time-frequency spectrogram in Fig. 4C highlights the dynamic frequency response during state transitions, with a clear shift in resonance frequency tracked over time. In particular, the resonance frequency transitions from ~140 kHz in the ON state to around 90 kHz in the OFF state. This shift occurs over a time span of ~0.2 s, as indicated by the dashed transition path. The amplitude, represented by the color gradient, shows a pronounced intensity (red regions) at the resonance frequencies, while other frequencies remain suppressed (blue regions). Leveraging this characteristic, the system enables straightforward and precise detection of phase transitions in single unit cell.

Achieving unit cell’s scalability and adaptability, we use a modular mechanoreceptor array (Fig. 4D), comprising 25 interconnected inductor-capacitor (LC) units, as further validated by the fabricated prototype shown in Fig. 4E. To ensure that each unit within the chain exhibits a distinct resonance frequency, we used different capacitances to design and fabricated it. The mechanical resonant characteristics of the nonreciprocal structural unit are influenced by the opening angle α between adjacent unit cells in each individual chain. In contrast, the resonant characteristics are not directly interconnected. Figure 4F demonstrates the tunable resonance characteristics of the chains (from C1 to C5), showcasing a systematic upward shift in resonance frequencies with increasing unit cell. Each chain has five distinct formants and the primary resonance frequency progresses from ~50 kHz (A5) to 140 kHz (A1). Although each chain is fabricated using the same method, their frequency response curves are not identical. These differences are summarized in table S1, including the maximum and minimum resonance peaks, mean, and coefficients of variation (defined as the ratio of standard deviation and mean), under both the open and closed states of the unit. The A2 unit at OFF stage exhibits the largest coefficient of variation at 0.072, while A1 unit shows the smallest at 0.019, representing 73.6% difference between the units. The highest amplitude peak is observed for C1 and C5 (~0.8 mV), and it slightly reduced in C2 (~0.68 mV). Amplitude variation of resonance peaks results from the units being connected by flexible ligaments, and the factors such as unit cell’s tension and printing errors cause frequency differences. These features show that the resonance characteristics are independently governed by the structural configuration. Precise control and identification of resonance peaks enable real-time sensing of structural phase transitions and their locations.

The mechanoreceptor’s resonant response to external forces is essential for assessing performance in information encoding and self-sensing robots. Figure 4G illustrates the relationship the opening angle α and the resonant frequency of mechanoreceptors in the C5 chain under both water-immersed and nonimmersed conditions. Across all mechanoreceptors (A1 to A5), the resonant frequency consistently increases with the opening angle, indicating a strong coupling between structural deformation and electromagnetic response, regardless of whether the system is in nonimmersed or water-immersed environment. Each mechanoreceptor exhibits a distinct resonant frequency, and the influence of dry and water environments on the resonant characteristics is minimal and nearly negligible. Notably, the resonant frequency differences among mechanoreceptors (e.g., A1 to A5) are preserved under both conditions, with A1 unit cell consistently exhibiting the largest resonant frequency range (from 95 to 193 kHz). During deformation, the unit cells exhibit unique resonant frequencies, and the differences in resonant frequencies between them are crucial for phase sensing. These results indicate that the structure’s electromagnetic properties are robust and resilient to environmental changes, allowing reliable frequency tuning and phase transition perception in both dry and wet conditions.

### Information encoding and computation by phase transition perception

**Fig. 5.**
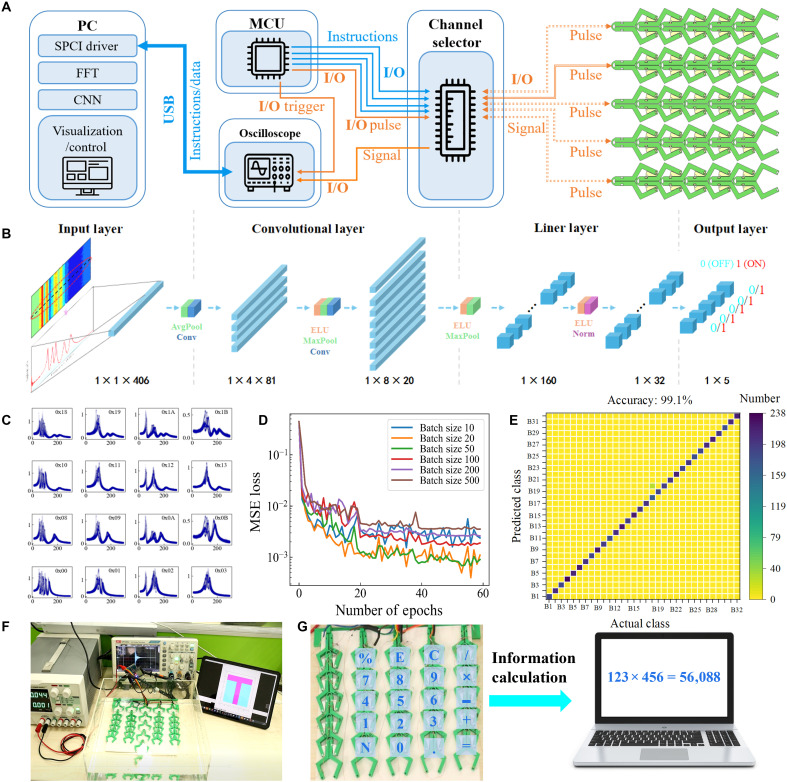
Applications of the mechanoreceptor array for information encoding and computation. (**A**) System architecture integrating a PC, microcontroller unit (MCU), oscilloscope, channel selector, and mechanoreceptor array. (**B**) Convolutional neural network (CNN) structure for real-time classification of sensor states. (**C**) Representative sample curve of mechanical sensor array: The horizontal axis in each subplot represents frequency (kilohertz), while the vertical axis denotes voltage (megavolts). The hexadecimal number in the upper right corner indicates the chain’s state, where ON represents 1 and OFF represents 0. For example, the hexadecimal value “0 × 05” corresponds to the binary sequence “00101,” meaning the unit states are OFF, OFF, ON, OFF, and ON, respectively. (**D**) Mean squared error (MSE) loss convergence for different batch sizes. (**E**) Confusion matrix illustrating the classification accuracy of 99.1% across 32 different bistable states. (**F**) Various states and their corresponding encoding patterns. (**G**) Demonstration of real-time information computation using the mechanoreceptor array as a keypad.

The mechanoreceptor array demonstrates precise in recognizing phase transitions. Leveraging this foundation, machine learning is adopted to enhance the accuracy and efficiency of phase transitions identification while significantly reducing the time and computational resources for signal extraction. Figure 5A presents a flowchart describing phase transition identification of the mechanoreceptor array. The microcontroller unit (MCU) generates a 5-μs square pulse via its input/output (I/O) to initialize the magnetic field in the chain’s coils, followed by high-impedance mode to enable free LC oscillation. A chip sequentially selects one of the five chains for signal acquisition, linking it to the MCU and an oscilloscope. The oscilloscope, triggered by the MCU, records the signal and transfers it to PC. The PC applies the convolutional neural network (CNN) to predict the chain state, visualizing results or integrating them into external computers.

It should be mentioned that the phase transition of bistable unit between the ON and OFF states is not discrete but continuous in actual nature. As shown in fig. S20, an intermediate state between ON and OFF is formed because of the extra force, and the gap between the magnetic core and the coil buckle increases, affecting the resonance frequency of the resonant circuit. Sufficient datasets can help neural networks identify these transitional states and output binary classification results as ON/OFF (see figs. S26 and S27). Machine learning (ML) model (Fig. 5B and fig. S25) is trained using 32,000 prelabeled data as 32 classifications (each unit has two states: ON or OFF, and each chain contains five units) by each chain. The model takes the spectrum as input (Fig. 5C) and predicts the states of five units as output. Notably, each chain within the mechanoreceptor array is trained independently. The resonant frequency signal and corresponding amplitudes are collected by stretching the unit cell of bistable element from the mechanoreceptor array. Dataset is randomly divided into training and test sets in a 7:3 ratio. The CNN model is trained using the training set, while the testing set is used to evaluate its room mean square loss and accuracy. We validated the optimal linear layer count to ensure prediction speed and minimize risk of overfitting during phase transition identification (fig. S28). The initial learning rate is set to 0.002 and decays by a factor 0.2 every 20 epochs. Six batch sizes (see Fig. 5D) are evaluated to determine their impact on model performance. In the training set, the loss function exhibits decreasing trend with increasing batch size, reaching a plateau beyond 50 (fig. S29). This means that a larger batch size can enhance phase recognition accuracy in the training set. However, the lowest loss is observed at batch sizes of 20 and 50 in the testing set. Both larger and smaller batch sizes result in degraded performance. Thus, we select the batch size of 50 for final model training to balance training stability and generalization.

Note that the ON-state frequency of A5 overlaps with the OFF-state frequency of A2 (see fig. S21). However, the classifier of our machine learning model is tolerant to such frequency variations and remains capable of accurate classification. The main reason is the frequency spectral contains sufficient redundant information. The system comprises five units, collectively exhibiting 10 resonant frequencies, yielding a total of 1024 unique spectral combinations. To investigate the effect of frequency differences on the classification accuracy, LC resonance differential equations with internal resistance are established and solved by the SciPy 1.0 package established by Oliphant (*61*). Machine learning can achieve greater than 95% accuracy when the *C_v_* of the system resonant frequency is less than 0.048 and the resonance peak frequency difference δ*_f_* exceeds 0.05 (see fig. S24F). Following model training, the trained neural network is used to identify phase transitions within the mechanoreceptor array. As demonstrated in Fig. 5E, the model achieves an exceptionally high classification accuracy of 99.1% in a confusion matrix for distinguishing different phase transition types.

To explore the practical feasibility of integrating the mechanoreceptor array with deep learning model for phase transition recognition, we investigate its versatile applications in information encoding, erasure, and computation by constructing a system similar to computer keyboard. Movie S7 and Fig. 5F illustrate the user manually actuates the bistable elements in the mechanoreceptor array, while ML performs real-time recognition of the sensor states. The array as a mechanical memory storage device successfully encodes and transmits the letters “WHUT” to the visualization system (see fig. S30), demonstrating precise programmability and control over the encoded states.

Additionally, movie S8 (fig. S31) and Fig. 5G describe the remarkable versatility and precision of the mechanoreceptor array in reprogrammable information processing, even under aqueous conditions. The external load effectively drives the physical transitions of the bistable elements, showcasing the robustness and adaptability of the system in water environment. This capability highlights the potential of the mechanoreceptor array for practical applications in harsh or variable conditions, such as underwater sensing and robots.

The mechanoreceptor array also is used for information computation, demonstrating its versatility beyond information encoding. To achieve this, the system interfaces with the built-in Windows calculator. Figure 5H (see movie S9 and fig. S32) shows that user interaction with mechanoreceptor array, where bistable elements are manually actuated to input numerical values. The array is configured as a keypad with distinct bistable elements assigned to digits (0 to 9), arithmetic operators (+, −, ×, and ÷), and functional keys (e.g., %, =, and C for enter). Real-time machine learning recognition detects sensor states, converting mechanical deformations into digital inputs, as demonstrated by the calculation of “123 × 456 = 56,088.” Actuation of different bistable elements enables accurate numerical encoding, demonstrating the potential of the array for tactile-based human-computer interaction, programmable input devices, and adaptive mechanical interfaces.

### Solitary wave–driven hydraulic propulsion in soft, self-sensing robots

**Fig. 6.**
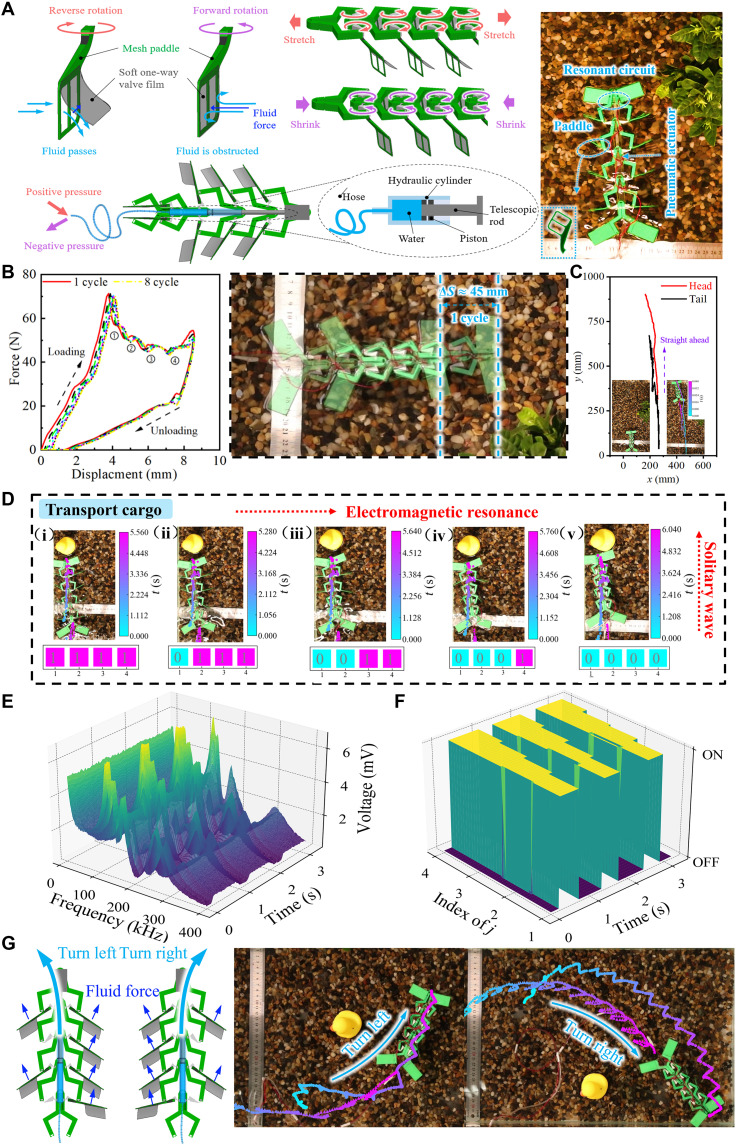
Solitary wave–driven hydraulic propulsion in soft and self-sensing robots. (**A**) Design and actuation mechanism of the soft robot. (**B**) Nonlinear mechanical response and solitary wave–induced propulsion. (**C**) Trajectories of robotic locomotion on the water. (**D**) Spatiotemporal mapping of robot deformation during cargo transport. (**E**) Frequency-time mapping of resonant voltage signals. (**F**) Binary decoding of unit activation states. (**G**) Schematic diagram illustrating the turning mechanism of the robot through asymmetric actuation.

The integration of phase transition dynamics with electromagnetic resonance in nonreciprocal mechanical metamaterials has enabled the development of soft robotic systems with self-sensing capabilities. Here, we present a water-floating robotic composed of four interconnected unit cells, eight paddle-like appendages, two hydraulic actuator, and four artificial mechanoreceptors based on electromagnetic resonance as shown in Fig. 6A and fig. S33. Rigid paddles tend to produce equal and opposite forces during forward and backward strokes, causing the robot to oscillate within a limited range without achieving net locomotion. To overcome this, soft paddle structures are designed to incorporate unidirectional valves. During the robot’s extension phase, the paddles move backward, and the valve membranes open under hydrodynamic pressure, allowing water to pass through and reducing the fluid inertial force acting against the robot. Conversely, the paddles move forward, the valves close, and fluid inertia is harnessed to generate propulsion during contraction. The propulsion mechanism is governed by solitary wave propagation, thus generating a cyclic, unidirectional motion that drives the robot forward.

To gain deeper insight into how the solitary waves can be harnessed for robotic crawling, we connect the external brake to the universal testing machine and conduct the cyclic loading-unloading experiments at the compression speed of 300 mm/min. Figure 6B characterizes the four bistable mechanical response (corresponding to four units) of the robots under cycle loading compression. The loading phase activates structural deformation uniformly, engaging its paddles to generate thrust. Instead, the solitary wave induces sequential paddle strokes through water upon unloading. This asymmetric actuation enables the robots to swim approximately Δ*S* ≈ 45 mm per cycle (~0.33 body lengths per cycle (BL/cycle); see table S4) (*62*–*67*). To this end, we programmed the robot for approximate linear motion through symmetrical propeller distribution and its trajectory is shown in Fig. 6C (see movie S10) over nine loading cycles. The head travels ~584 mm along the longitudinal direction with less than 60-mm lateral deviation, indicating an effectively linear trajectory.

In many scenarios, robots are required to transport cargo reliably across dynamic environments, demanding both robust actuation and real-time feedback. Driven by solitary waves and hydraulic actuation, the robot advances ~40 mm while transporting cargo (see Fig. 6D and movie S11). Electromagnetic resonance provides spatiotemporal insight unit deformation and accurately real-time identifies transition waves during locomotion by machine learning. As shown in Fig. 6E, each unit cell exhibits distinct voltage peaks (~6 mV) at characteristic frequencies when transitioning between open and close states. These resonant responses are discretized in Fig. 6F, where the ON/OFF states of the four mechanoreceptors are clearly detected over time, matching the activation sequence. Thus, the resonance-based mechanoreceptor can accurately and continuously track structural dynamics during solitary wave–driven motion.

Besides forward propulsion, the robotic motion direction can also be controlled by adjusting the numbers of the paddles and reconfiguring their spatial locations. When the moment produced by the paddles at opposite sides is unequal, an asymmetric motion appears, and it pushes the robot turn toward. Figure 6G illustrates this steering capability, and the imbalanced fluid forces induce a smooth leftward turn. The robot takes a left turn of ~65° (see movie S12) and right turn of ~58° (see movie S13). The velocity profile of the robot’s head and tail in both longitudinal and lateral directions during locomotion is plotted in fig. S35. The forward velocity and accelerate peak are 187.5 mm/s and 1.3 m/s^2^, respectively, demonstrating the system’s high dynamic responsiveness under cyclic soliton-hydraulic actuation.

## DISCUSSION

This work introduces an innovation approach for phase transition perception and nonlinear wave manipulation through the nonreciprocal metamaterials integrated with electromagnetic resonance. Inspired by the kinematic mechanisms in ostrich locomotion, the nonreciprocal metamaterials combining soft and rigid components are designed to achieve unidirectional propagation of topological solitons through eliminating the maximum structural energy barrier without external stimuli. The wave velocity could be customized through precise design of the bistable unit. By harnessing electromagnetic resonance and deep learning model, the system achieves a phase transition identity accuracy of 99.1%. The modular mechanoreceptor arrays enable robust phase transition perception for information encoding, erasure, and programmable information computation.

In addition, the designed nonreciprocal structure is adopted to construct a soft robotic system powered by the interplay of topological solitons and hydraulic actuation. The robot translates solitary waves into the power that directed mechanical motion, enabling robust locomotion in the water. By orchestrating asymmetric paddle strokes through sequential structural deformation, the robot achieves forward propulsion of ~45 mm per actuation cycle (~0.33 BL per cycle). The solitary wave arises spontaneously during unloading, highlighting the intrinsic mechanical intelligence embedded within the metamaterial design.

The mechanoreceptors constructed by the metamaterial and electromagnetic resonances can continuously monitor the deformation state. These mechanoreceptors allow real-time mapping (the time resolution of mechanoreceptors is ~660 μs) of phase transitions and unit activations, i.e., phase transitions are continuously tracked and visualized with the structural motion or deformation. This capability enables both adaptive control and early detection of mechanical failure, such as premature closure due to structural instability. The combination of nonlinear wave dynamics and distributed sensing paves the way for autonomous, self-aware soft robots capable of operating reliably in complex and unstructured environments.

## MATERIALS AND METHODS

### Sample preparation of the nonreciprocal metamaterials

All samples were printed using a 3D printer via FDM 3D printing with a precision of ~0.2 mm. The printed materials were high-performance PETG with a Young’s modulus *E*_s_ = 1.107 GPa (fig. S1) and TPU with a Mooney-Rivlin constitutive model (*C*_10_ = 0.122 MPa, *C*_01_ = 3.556 MPa, and *D*_1_ = 0.0181 MPa^−1^). The coil (Beijing Zhongci Smart Sensing Technology Co., China) was wound using 0.3-mm copper wire around a 2-mm-diameter metal rod, with ~10 turns per layer and a total of four layers. The magnetic core had a relative permeability of 1600 H/m, a diameter of 1.5 mm, and a length of 8.92 mm. Five distinct signal chains were constructed using capacitors of different capacitances: 0.22, 0.33, 0.47, 0.68, and 1 μF, respectively. These varying capacitances established different resonance frequencies, enabling the neural network to distinguish signals originating from individual unit cells.

### Measurement of the quasi-static mechanical behavior test

The uniaxial tension tests were performed at room temperature 25° using a universal mechanical testing machine (DN-W2kN, China) from which the displacement-force curves were obtained. The force sensor of the machine had a capacity of 20 N and a resolution of 0.01 N. These stiffness values of soft hinges are measured by the force sensor (HANDPI-10 N, Yueqing HANDPI Instrument Co. Ltd., China). During conducting experiments, the loading-unloading speed of all specimens were set to 2 mm/min. we used the Sony camera (Sony A7C, Japan) to capture the deformation process of the structure during the experimental procedure. The deformed displacement field was subsequently analyzed using MatchID 2D software (Digital Image Correlation, Belgium). Each unit was equipped with a tracker, allowing measurement of the displacement of each cell.

### Measurement of the dynamic mechanical behavior test

To experimentally demonstrate the nonreciprocity of metamaterials, we use a 1 × 40 row samples to study the dynamic mechanical behavior, and their dynamics are recorded by a camera. We then analyzed our experiments by tracking the embedded particles (i.e., red dot) on the surface of structure and calculate the propagation process of the nonlinear transition waves using python codes.

### Computational and analytical modeling

The commercial ABAQUS 2023 software was used to perform the FEA. The maximum strain of PETG was ~0.03 during the metamaterials’ tensile process. Therefore, a linear elastic constitutive relationship (Young’s modulus *E*_s_ = 1.107 GPa, Poisson ratio *v*_s_ = 0.3) was used to simplify the simulation of PETG materials. The incompressible Mooney-Rivlin hyperelastic constitutive relationship was used to model the deformations of the TPU. The friction coefficient was set as 0.1, and the general hard contact was used to perform this simulation. A refined mesh and CPE4R elements were used to enhance the accuracy of the numerical simulation. The clamped-clamped condition has been realized by fixing the nodal points of one end of the structure, while the nodal points of the moving end have freedom to move along the axial direction of the chain end. In the first and last models, we tied the end nodal points at each end to a reference point and applied displacement loads. Then, we simulated the propagation of nonlinear waves in the chain based on a preliminary static simulation. Based on these results, we used the statics and energy method to establish the kinematic theory of the multibody architecture (see Supplementary Text S2). Then, we derive the governing equations of the chain and solving them numerically to characterize the transition wave propagation using Lagrange’s equations (see Supplementary Text S3).

### Experimental setups for the signal measurements

An ESP32 MCU was used to generate 5-μs square wave pulses (SDG1022X, SIGLENT) via its I/O ports, establishing initial magnetic fields in the coils of the chains. Subsequently, the I/O ports were set to high-impedance states, allowing the LC resonant circuits, composed of the coils and capacitors within the chains, to oscillate freely. To sequentially acquire signals from the five chains, channel selection was managed using a CD74HC4067 multiplexer, connecting the selected chain to both the MCU and an oscilloscope. Before pulse transmission, the MCU emitted a trigger signal to initiate data acquisition by the oscilloscope. A computer, interfaced with the oscilloscope (UNIT UPO1202S-E) via the Standard Commands for Programmable Instruments (SCPI) protocol, was used to configure oscilloscope parameters and retrieve data. Upon acquiring the free oscillation signals, the computer performed a Fourier transform on the data and input the transformed data into a CNN to predict the states of the chains.

### Data acquisition

Python scripts were developed using pyvisa, numpy, and pyserial libraries to automate data acquisition. An ESP32 microcontroller was used to excite unit oscillations, and signal acquisition is performed via oscilloscope connected through the SCPI protocol at a sampling rate of ~32 samples per second. System configuration details were provided in Fig. 5A. For each state, 1000 samples were collected over ~30 s. During data collection, the chain units were held in the predefined ON or OFF states, while combinations of tensile, compressive, bending, and torsional deformations of varying magnitudes were applied to induce deviations from ideal configurations. These perturbations were introduced to emulate experimental uncertainties and enhance the robustness of the neural network.

### Design of self-sensing robots

Given that the bistable structure cannot autonomously revert to its extended state after contraction and requires external energy for resetting, we used a hydraulic system as the actuation mechanism. The robot’s extension necessitated an actuation stroke of ~70 mm, and the available space for installing the hydraulic actuator is limited. Therefore, we selected a 1-ml plastic syringe as the hydraulic actuator, connecting it to a hydraulic pump via flexible tubing. When the hydraulic pump applied positive pressure, the actuator extends, resetting the robot to its stretched configuration. Notably, due to the insufficient rigidity of the plastic syringe plunger, there is a risk of buckling during the robot’s expansion. To mitigate this, we reinforced the plunger by embedding a 1-mm-diameter steel wire within it, preventing instability and ensuring reliable operation.

## Supplementary Materials

### The PDF file includes:

Supplementary Text Figs. S1 to S35 Tables S1 to S4 Legends for movies S1 to S13 Legend for code S1

### Other Supplementary Material for this manuscript includes the following:

Movies S1 to S13

Code S1
