# Supplementary material for: Phase transitions perception in nonreciprocal mechanical metamaterials through electromagnetic resonance
Source: Sci Adv. 2025 Sep 12;11(37):eady1211. doi: 10.1126/sciadv.ady1211 (PMC12429039; doi:10.1126/sciadv.ady1211)
Supplement: Supplementary file 1 — Supplementary Text Figs. S1 to S35 Tables S1 to S4 Legends for movies S1 to S13 Legend for code S1 [file sciadv.ady1211_sm.pdf]

Science Advances
AAAS

Supplementary Materials for

Phase transitions perception in nonreciprocal mechanical metamaterials through electromagnetic resonance

Yun Deng *et al.*

Corresponding author: Zhixin Huang, huangzhixin1802@163.com; Ying Li, bitliying@bit.edu.cn

Sci. Adv. **11**, eady1211 (2025)
DOI: 10.1126/sciadv.ady1211

The PDF file includes:

Supplementary Text
Figs. S1 to S35
Tables S1 to S4
Legends for movies S1 to S13
Legend for code S1

Other Supplementary Material for this manuscript includes the following:

Movies S1 to S13
Code S1

Text S1 Design strategy of the non-reciprocal mechanical metamaterials

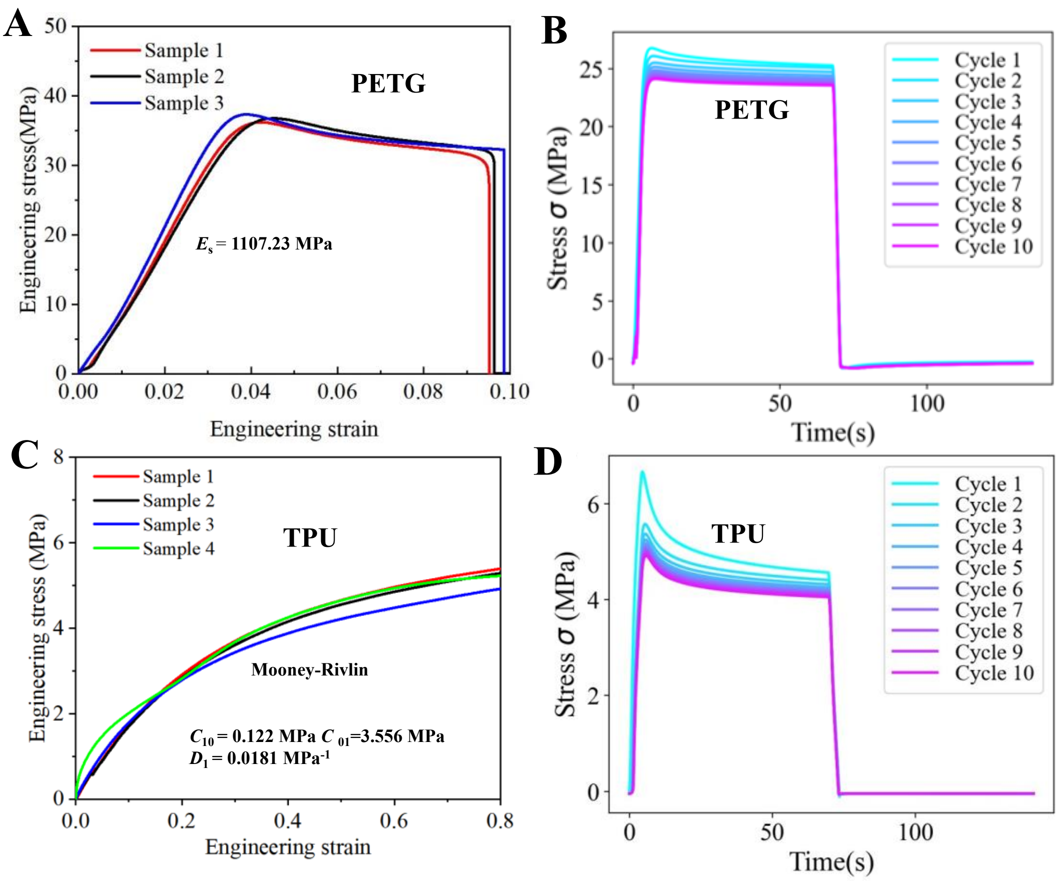

Fig. S1 Constitutive model of rigid and soft polymers. (A) The engineering strain-stress curves of PETG with a linear elastic modulus of 1107MPa; **(B)** The instantaneous and relaxation data of PETG under 10 cycles; **(C)** The engineering strain-stress curves of TPU with Mooney-Rivlin constitutive model (C_{10}=0.122MPa C_{01}=3.556MPa D_1=0.0181MPa^{-1}). **(D)** The instantaneous and relaxation data of TPU under 10 cycles.

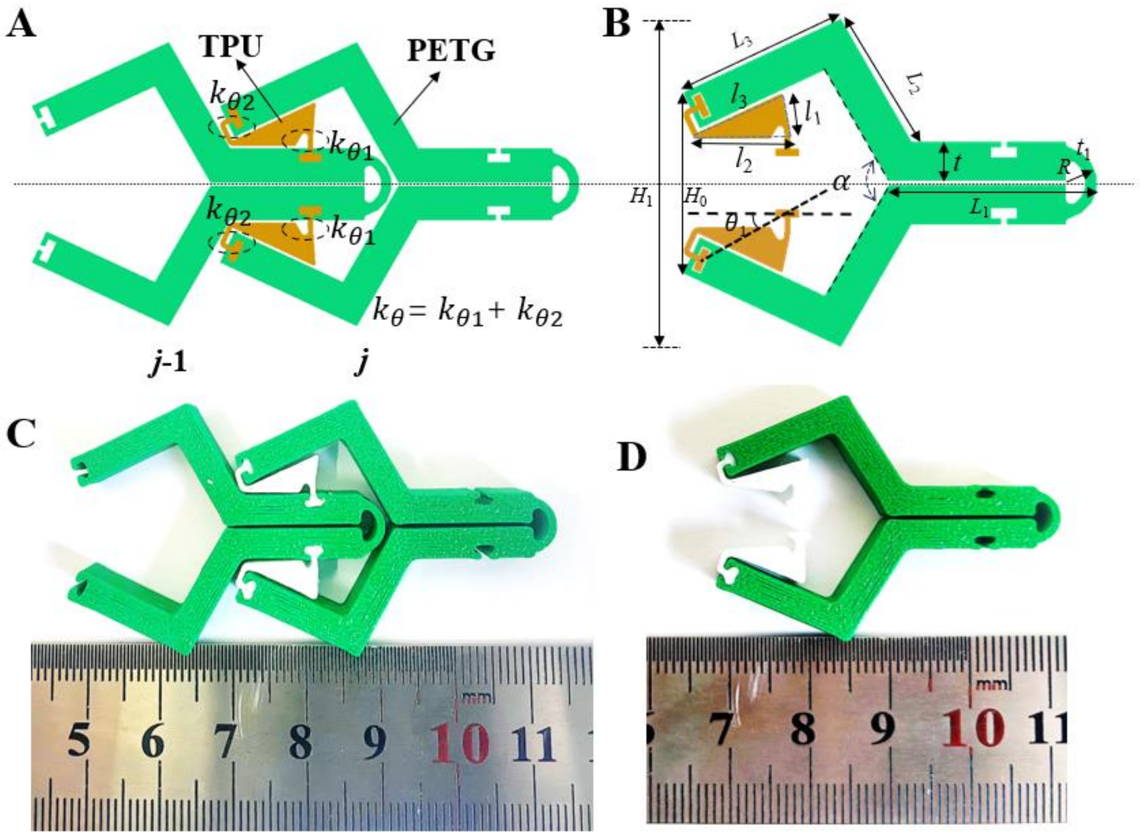

Fig. S2 Design of the non-reciprocal mechanical metamaterials. (A, C) The designed and optical image of structural model composed of PETG and TPU materials. **(B, D)** The geometric design parameters and optical image of unit cell.

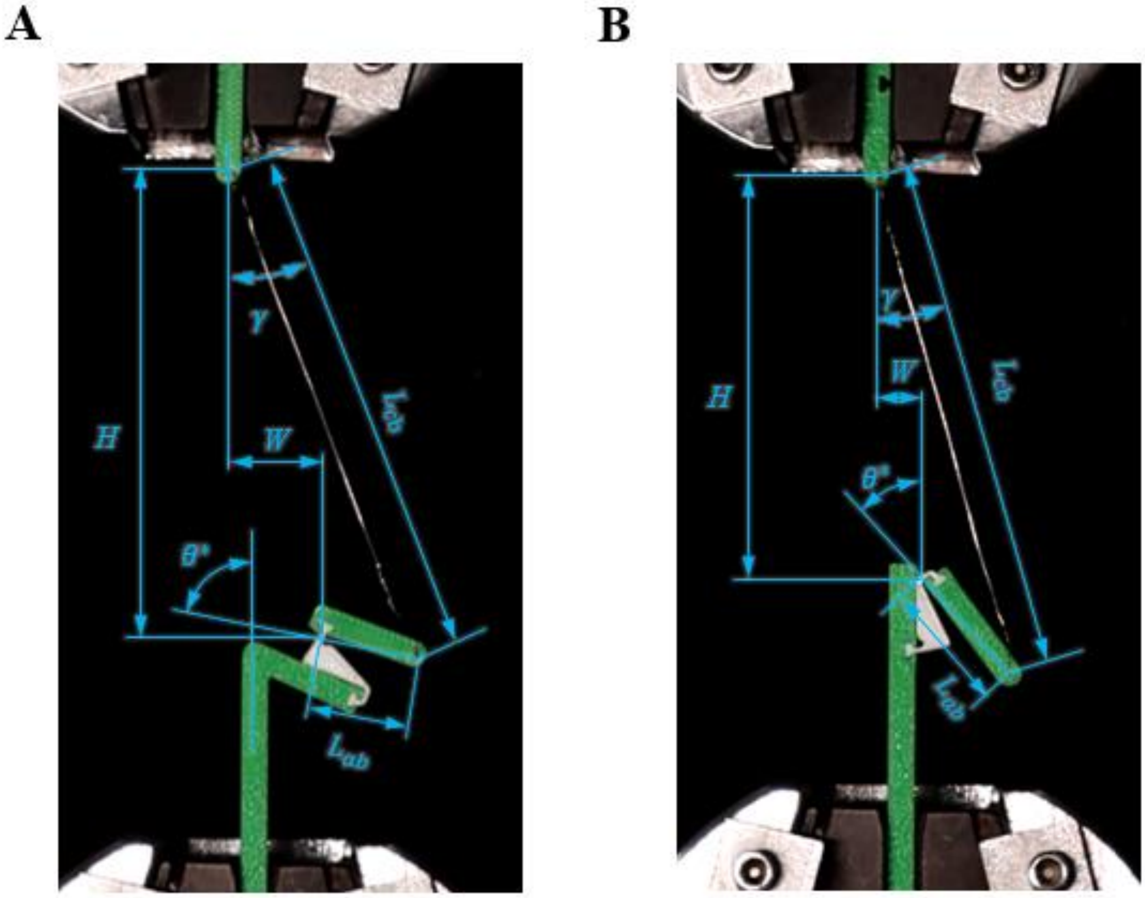

Fig. S3 The schematic diagram of k_θ measurement: **(A)** $k_{\theta 1}$(L_{ab} = 14.2mm, L_{cd} = 74.7mm, W=12.9mm, H_0= 66.9mm, θ_0^*=1.37rad, γ_0 = 0.367rad); **(B)** $k_{\theta 2}$ (L_{ab} = 18.8mm, L_{cd} = 74.7mm, W=7.72mm, H_0= 57.9mm, θ_0^*=0.735rad, γ_0 = 0.276rad).

The geometric equation of this system in Fig. S3 could be described in

$$L_{\mathrm{ab}} \sin\left(\theta^{*}\right) + W - L_{\mathrm{cb}} \sin\left(\gamma\right) = 0 \tag{1}$$

$$L_{\mathrm{ab}} \cos\left(\theta^{*}\right) + \left(H_{0} + \Delta H\right) - L_{\mathrm{cb}} \cos\left(\gamma\right) = 0 \tag{2}$$

The rotational spring of k_{θ} could be calculated as:

$$k_{\theta} = \sum_{i=1}^{2} k_{\theta,i} = \sum_{i=1}^{2} \frac{\Delta M_{i}}{\Delta \theta_{i}} \tag{3}$$

$$\Delta M_{i} = \frac{\Delta F_{i}}{\cos\left(\beta_{i}\right)} L_{\mathrm{ab},i} \sin\left(\alpha_{i} - \beta_{i}\right) \tag{4}$$

$$\Delta \theta_{i} = \theta^{*} - \theta_{0}^{*} (i = 1, 2) \tag{5}$$

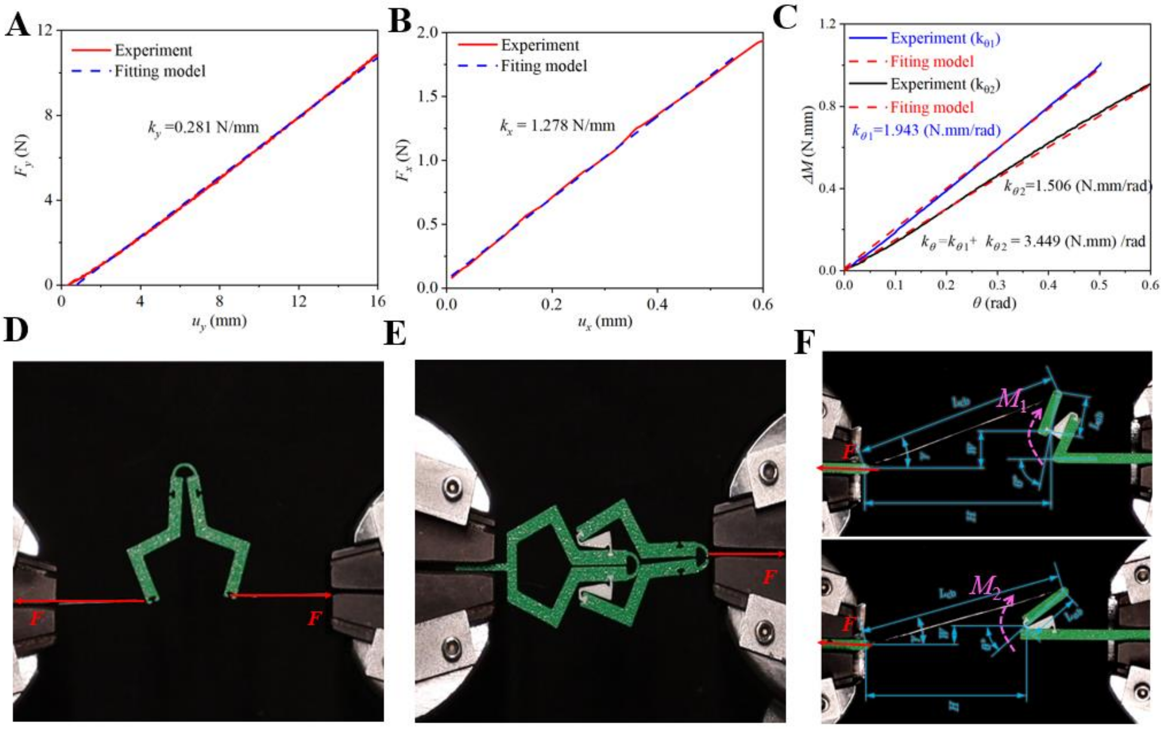

Fig. S4 Experiment results of the rigid body system constrained by hinges the force sensor (HANDPI-10N, Yueqing HANDPI INSTRUMENTS CO.,LTD). **(A-B)** and **(D-E)** two grounded springs k_y, k_x in the upper end; **(C-F)** the rotational spring k_θ at bottom side.

Text S2 Kinematics theory for multi-body architecture

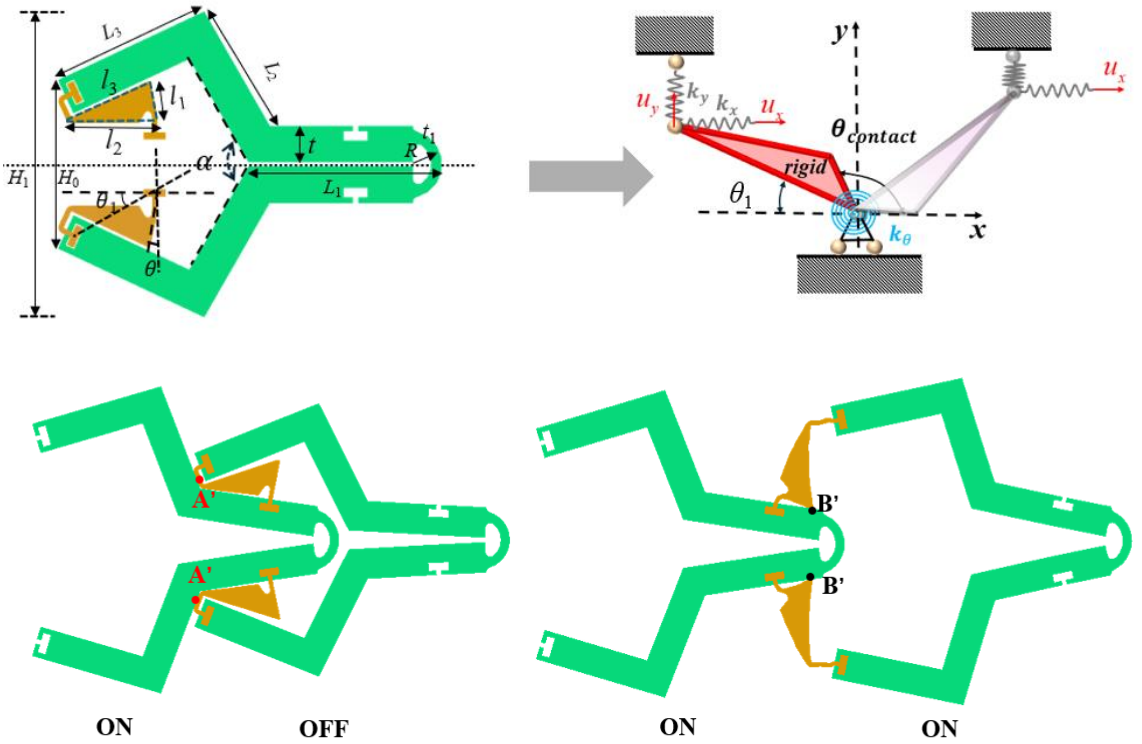

Fig. S5 Simplified multibody kinematic model of metamaterials.

The multi-body architecture of our concept display reflection symmetry about x axes. We thus develop here a theoretical model of its half and have chosen the rotation angle θ_1 as the independent variable. The total elastic potential energy of entire structure includes four parts:
The elastic potential energy of rotating spring k_θ :

$$U_\theta = \frac{1}{2} k_\theta \left(\theta_1 - \theta_0\right)^2 \tag{6}$$

The elastic potential energy of vertical spring k_y :

$$U_y = \frac{1}{2} k_y l_2^2 \left(\sin\theta_1 - \sin\theta_0\right)^2 \tag{7}$$

The elastic potential energy of horizontal spring k_x :

$$U_x = \frac{1}{2} k_x \left(l_2 \left(\cos\theta_1 - \cos\theta_0\right) - u_x\right)^2 \tag{8}$$

The contact takes place only if $\theta > \theta_c + \theta_b$, hence, we introduce the Heaviside function δ to express the U_{contact} as:

$$U_{\text{contact}} = \frac{1}{2} \eta_c \delta(\theta_1) \tag{9}$$

$$\delta(\theta) = \begin{cases} 0 & \theta_1 < \theta_c + \theta_b \\ \left(\theta_1 - \theta_c - \theta_b\right)^2 & \theta_1 \geq \theta_c + \theta_b \end{cases} \tag{10}$$

Where η_c is a sufficiently large parameter with a unit of N.m;
In this case, the total potential of the system is given by:

$$\Pi = U_\theta + U_y + U_x + U_{\text{contact}} - \int_0^{u_x} F_x \mathrm{d}u_x \tag{11}$$

The governing equations can be obtained by applying the Principle of Minimum Potential Energy:

$$\frac{\partial \Pi}{\partial \theta_1} = 0 \tag{12}$$

The force-displacement curve F_x - u_x can be solved by using the Pseudo Arc-length method with θ_1 being the control parameter as shown in Figure S5.

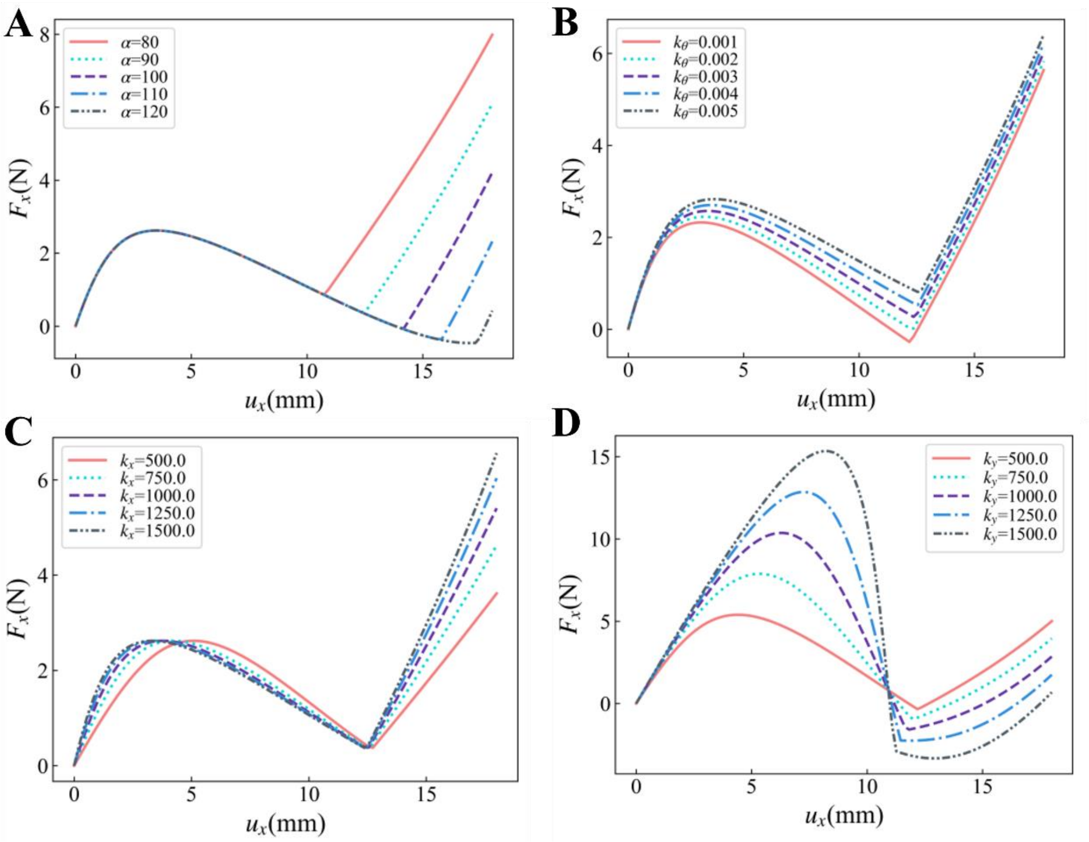

Fig. S6 The theoretical displacement-force curves of non-reciprocal metamaterials: Evolution of the *x*-direction force-displacement curve with varying (A) the opening angle α ; **(B)** the rotation spring k_θ ; **(C)** the horizontal spring k_x ; **(D)** the vertical spring k_y .

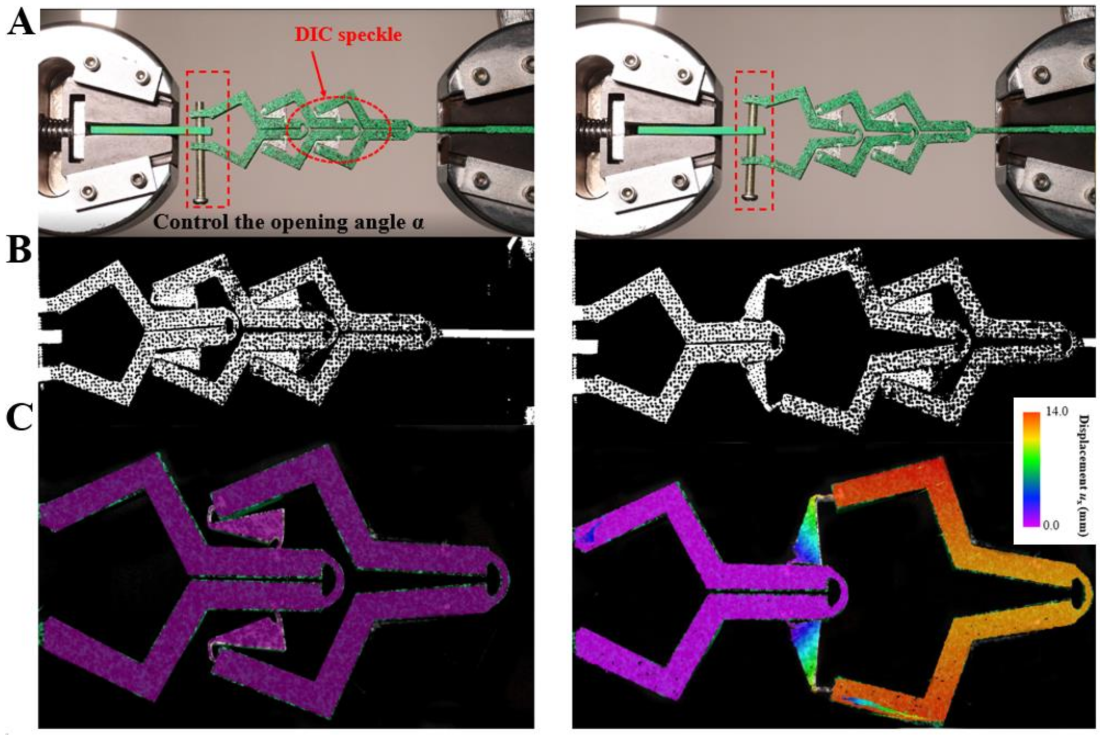

Fig. S7 The experimental setup for mechanical testing of the metamaterial structure: (A) the sample is clamped at both ends, and the opening angle α is controlled. Digital image correlation (DIC) speckle pattern is applied to track deformation; **(B)** Binary DIC speckle pattern image of the metamaterial under tensile deformation is used to extract displacement and strain distribution; **(C)** Displacement distribution analysis based on DIC measurements.

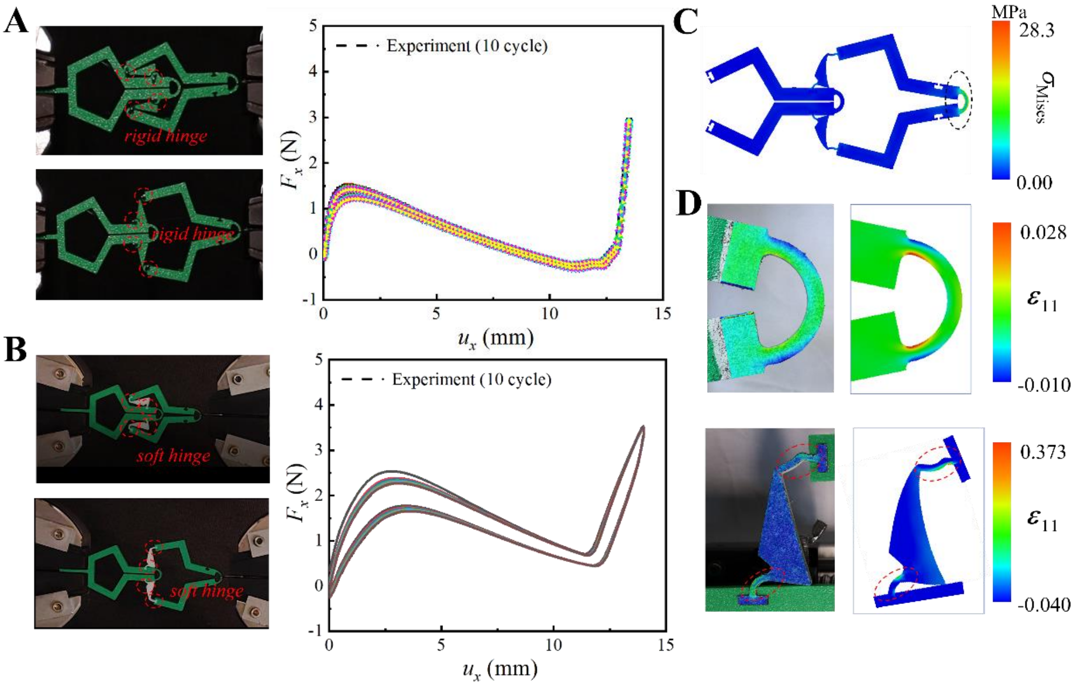

Fig. S8 Comparison of mechanical response between rigid hinges and soft hinges under 10 cycles. The force-displacement F_x-u_x of metamaterials with **(A)** rigid hinges; **(B)** soft ligaments;**(C)** the von-Mises stress of metamaterials with soft ligaments; **(D)** Comparison of experimental (DIC) and simulated strain ε_{11}.

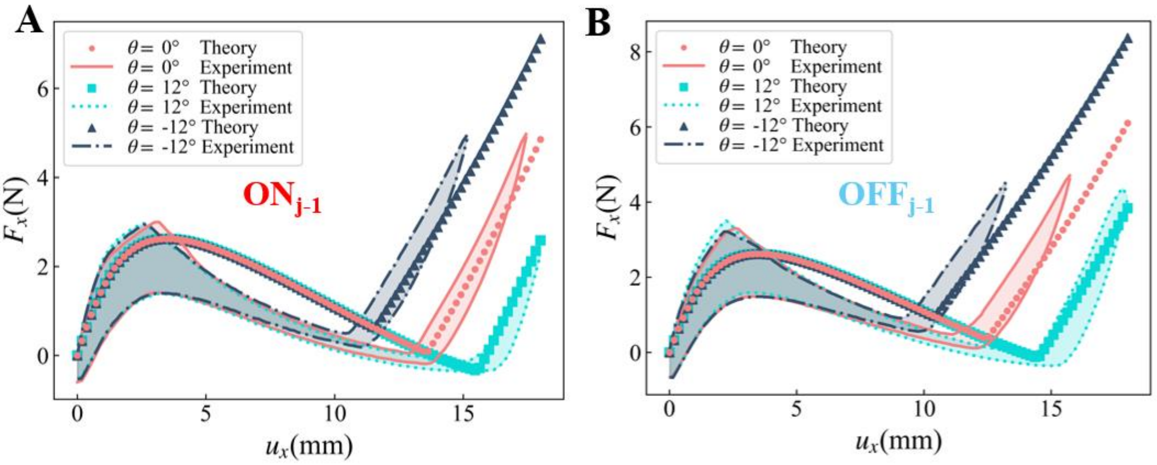

Fig. S9 The theoretical and experimental displacement-force curves of non-reciprocal metamaterials with varying the opening angle α and the rotation angle θ:(A) ON; **(B)** OFF.

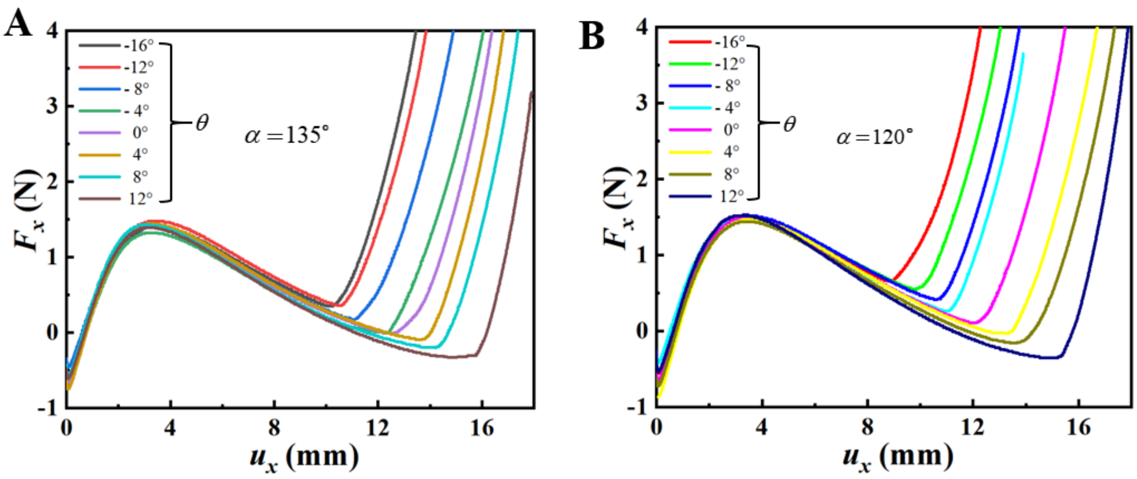

Fig. S10 Experimental load-displacement curves under different geometric parameters during unloading conditions. Force-displacement (F_x-U_x) curves for structures with varying initial angles under different geometric configurations in **(A)** ON and **(B)** OFF.

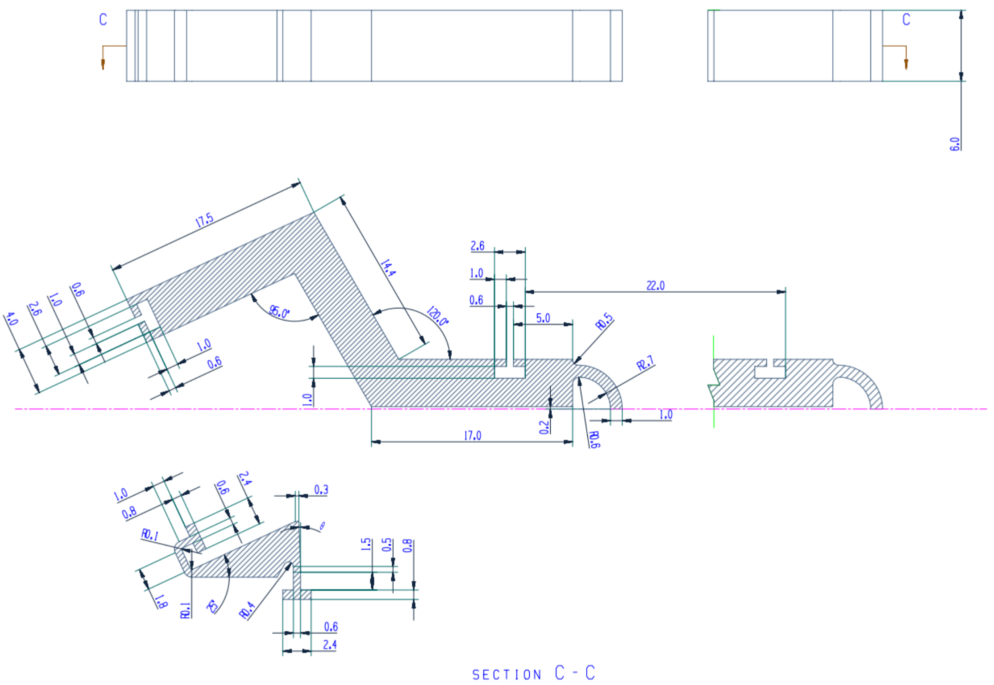

Fig. S11 The specific geometric parameters of metamaterials

Text S3 Non-reciprocal hinges dynamical model

We begin by deriving the governing equations of the metamaterials in the chain and solving them numerically to characterize the transition wave propagation. To this end, we use the non- conservative Lagrange equations of the system is given by:

$$L = \frac{1}{2} m_i (\frac{\mathrm{d}u_i}{\mathrm{d}t})^2 - \left(U_{\mathrm{left},i\text{-}1}(u_i - u_{i-1}) + U_{\mathrm{right},i}(u_{i+1} - u_i) \right) \tag{13}$$

The elastic potential energy of the system is determined by the kinematic equation (11) mentioned above:

$$U_{\mathrm{left},i-1}(u_i - u_{i-1}) = \Pi\left(u_i - u_{i-1}\right) \tag{14}$$

$$U_{\mathrm{right},i-1}(u_{i+1} - u_i) = \Pi\left(u_{i+1} - u_i\right) \tag{15}$$

The non-conservative dissipation of the system includes damping dissipation and friction dissipation:

$$R_1 = -\mu_k N \operatorname{sgn}(\dot{u}_i) \tag{16}$$

$$R_{\mathrm{left},i-1} = \frac{1}{2} \xi (\dot{u}_i - \dot{u}_{i-1})^2 \tag{17}$$

$$R_{\mathrm{right},i} = \frac{1}{2} \xi (\dot{u}_{i+1} - \dot{u}_i)^2 \tag{18}$$

$$R = R_1 + R_{\mathrm{left},i-1} + R_{\mathrm{right},i} \tag{19}$$

Where m_i is the mass of the metamaterials; $U_{\mathrm{left},i\text{-}1}(u_i\text{-}u_{i\text{-}1})$ represents the elastic potential energy provided by the (i-1)-th unit on the left to the i-th unit; $U_{\mathrm{right},i\text{-}1}(u_{i+1}\text{-}u_i)$ represents the elastic potential energy provided by the (i+1)-th unit on the left to the i th unit; μ_k is the friction coefficient and N represents positive pressure. R_1 is the ground friction. $R_{\mathrm{left},i\text{-}1}$ and $R_{\mathrm{right},i\text{-}1}$ is the damping force.

According to the non-conservative Lagrangian dynamic equation:

$$\frac{\mathrm{d}}{\mathrm{d}t}(\frac{\partial L}{\partial \dot{u}_i}) - \frac{\partial L}{\partial u_i} = -\frac{\partial R}{\partial \dot{u}_i} \tag{20}$$

from which the equilibrium equation can be derived using (13) as

$$m\ddot{u}_i + F_{x,L}(u_i - u_{i-1}) + \xi(\dot{u}_i - \dot{u}_{i-1}) - F_{x,L}(u_{i+1} - u_i) - \xi(\dot{u}_{i+1} - \dot{u}_i) + \mu_k N \operatorname{sgn}(\dot{u}_i) = 0 \tag{21}$$

Eq.(21) represents the discrete governing equation of the system, which we numerically solve using the 4th order Runge-Kutta method.

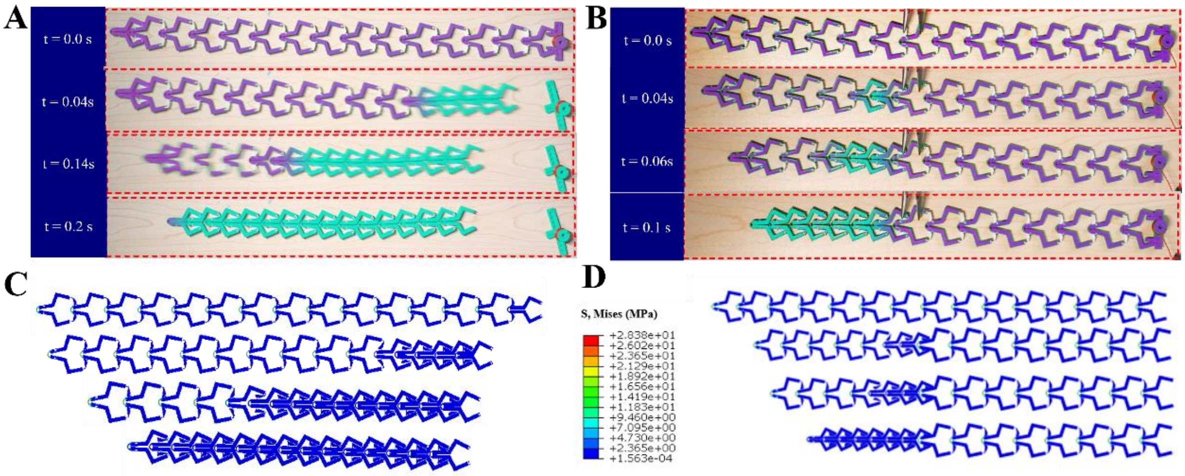

Fig. S12 Experimental and numerical photographs of propagation with the transition wave consisting of 15 cells. (A) Experiment: from right to left; **(B)** Experiment: from left to right; **(C)** Finite element model: from right to left; **(D)** Finite element model: from left to right.

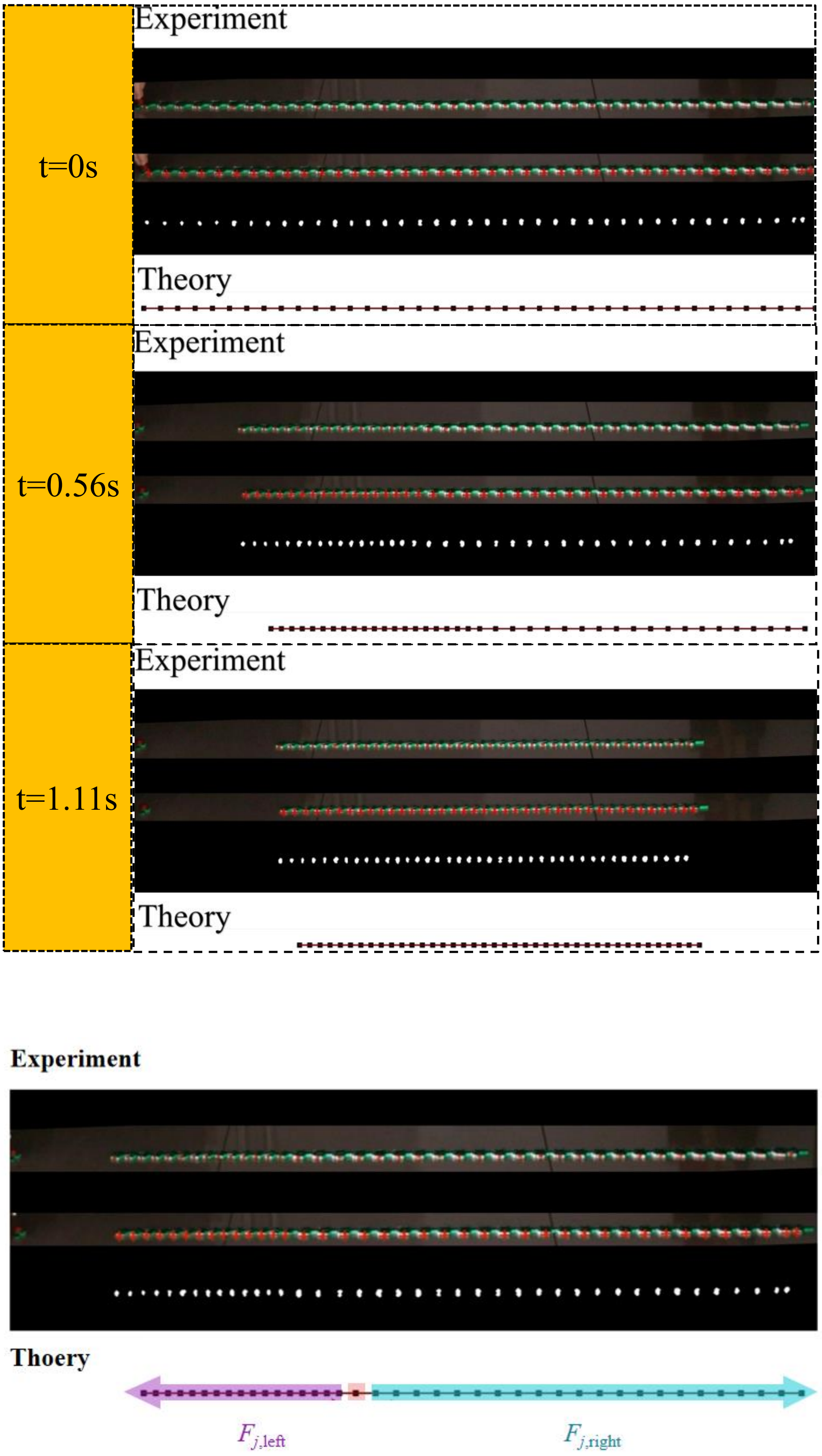

Fig. S13 Experimental and theoretical photographs of propagation with the transition wave consisting of 40 cells.

$$F_{j,\mathrm{left}} = \sum_{i=1}^{j} m_i (g\mu_k \,\mathrm{sgn}(v_i) + \dot{v}_i) \tag{22}$$

$$F_{j,\mathrm{right}} = \sum_{i=j}^{n} m_i (g\mu_k \,\mathrm{sgn}(v_i) + \dot{v}_i) \tag{23}$$

Where F_j is dragging force of the j-unit cell.

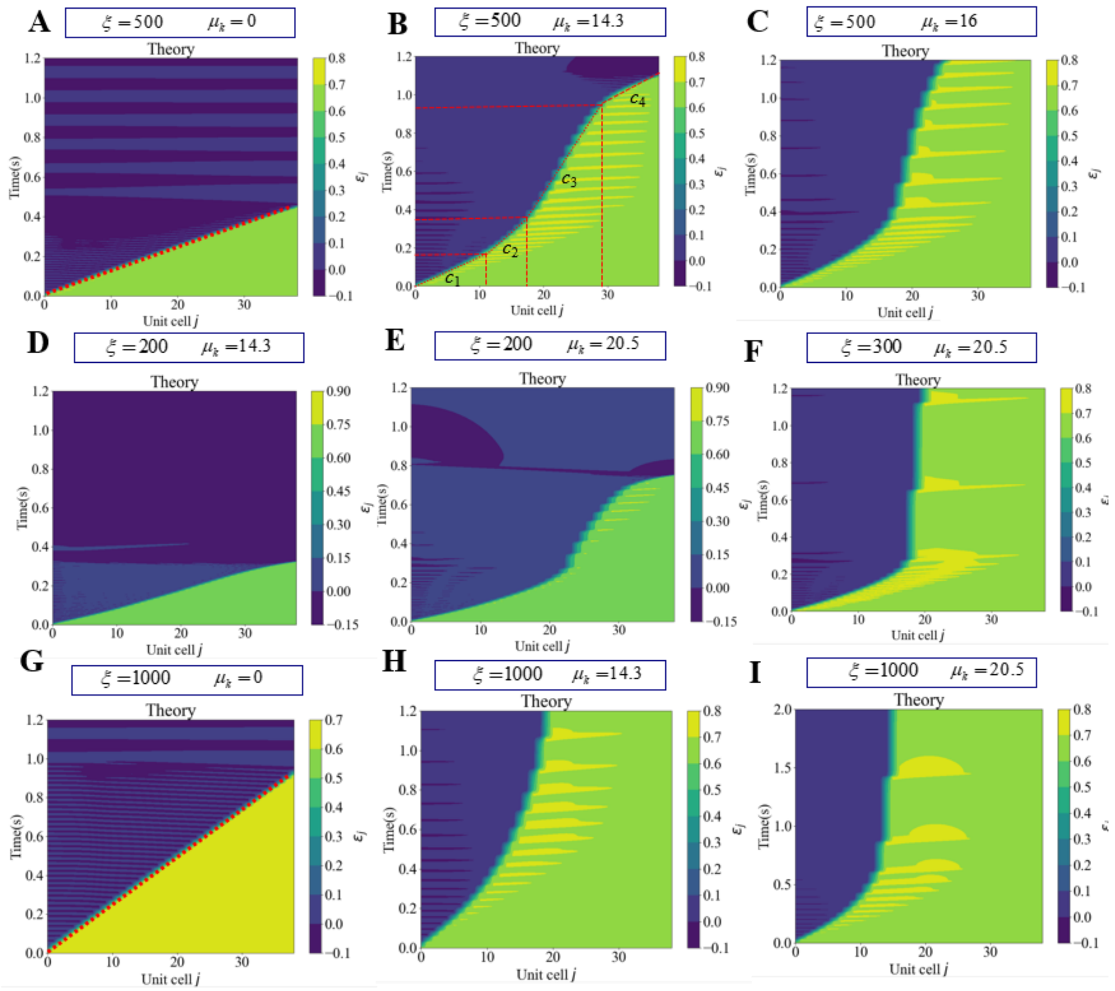

Fig. S14 Theoretical model on the evolution of the strain of each bistable unit in homogeneous transmission under different structural damping and ground friction. (A) ξ=200 μ_k = 0; **(B)** ξ=500 μ_k = 14.3; **(C)** ξ=500 μ_k = 16; **(D)** ξ=200 μ_k = 14.3; **(E)** ξ=200 μ_k = 20.5; **(F)** ξ=300 μ_k = 20.5; **(G)** ξ=1000 μ_k = 0; **(H)** ξ=1000 μ_k = 14.3; **(I)** ξ=1000 μ_k = 20.5.

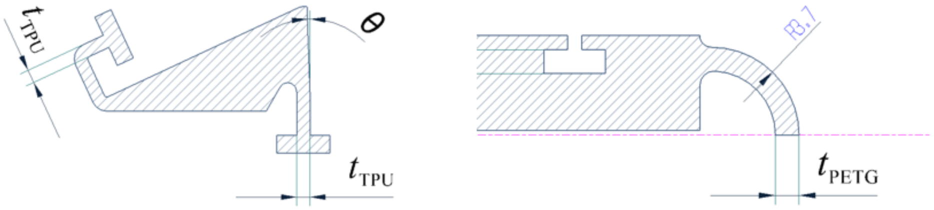

Model	θ(°)	t_{PETG}(mm)	t_{TPU} (mm)
Fast	-4	1.0	0.4
Medium	0	0.85	0.4
Slow	8	0.7	0.4

Fig. S15 The structural geometric parameters with customizable wave velocity.

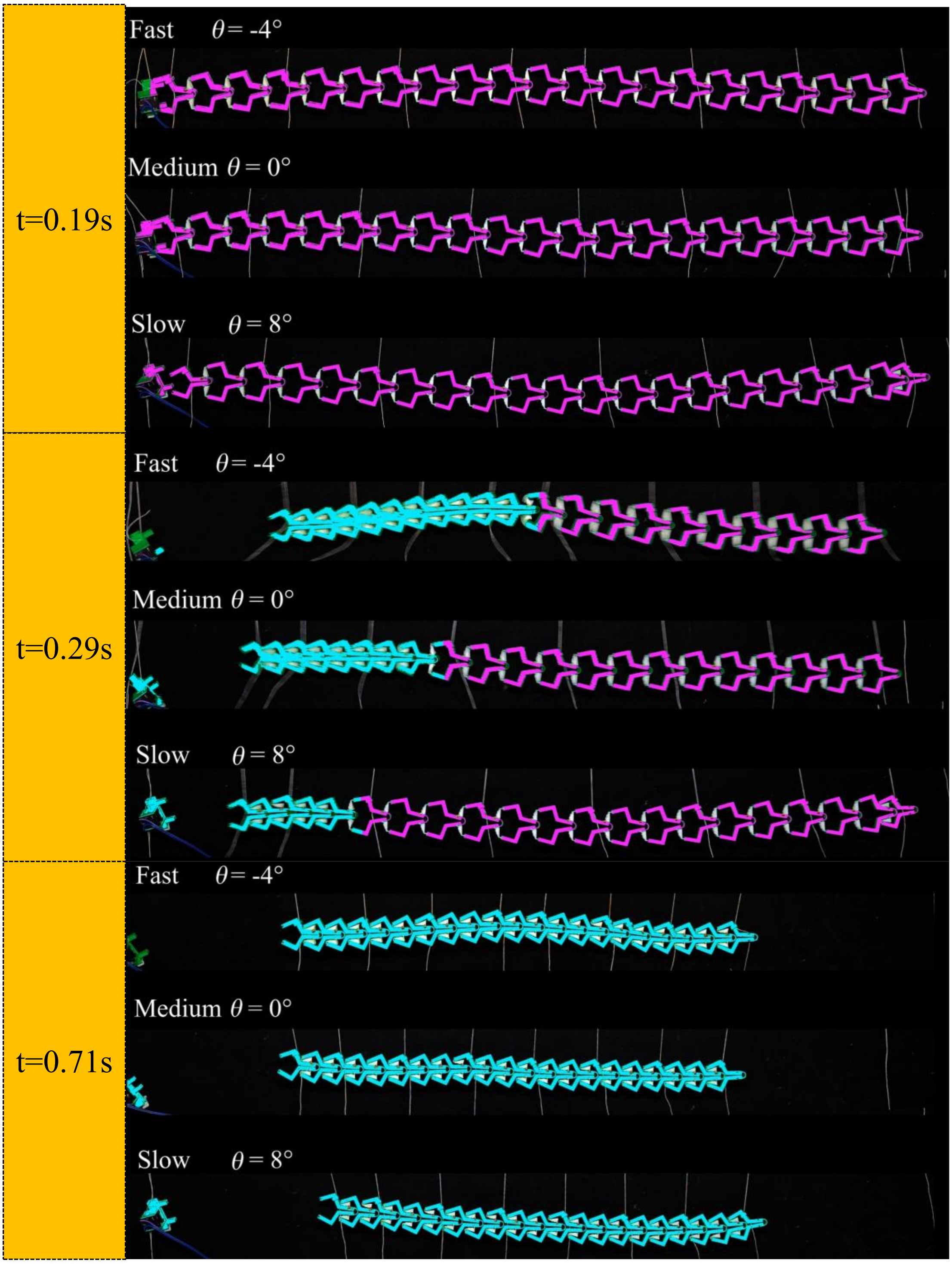

Fig. S16 The non-friction experimental photographs of propagation with the different transition wave velocity consisting of 20 cells.

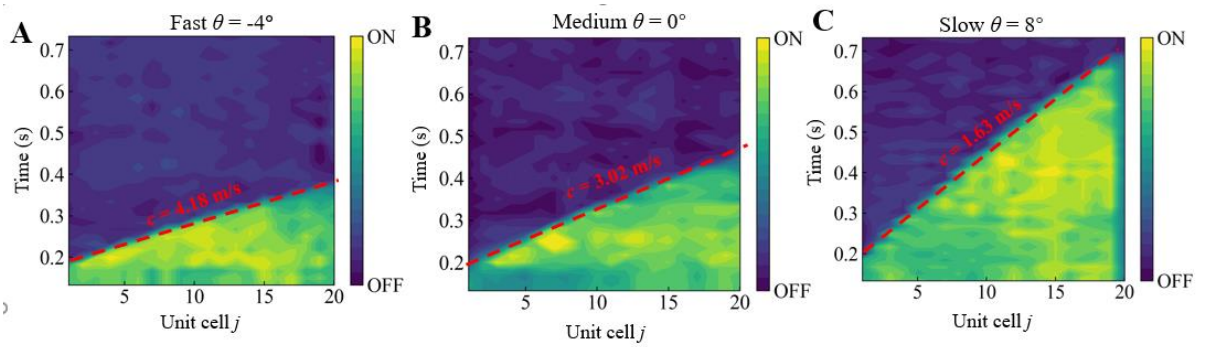

Fig. S17 The non-friction experimental results on the evolution of the state (ON or OFF) of each bistable unit with different wave velocity:**(A)** c = 4.18 m/s; **(B)** c = 3.02 m/s; **(C)** c = 1.63 m/s.

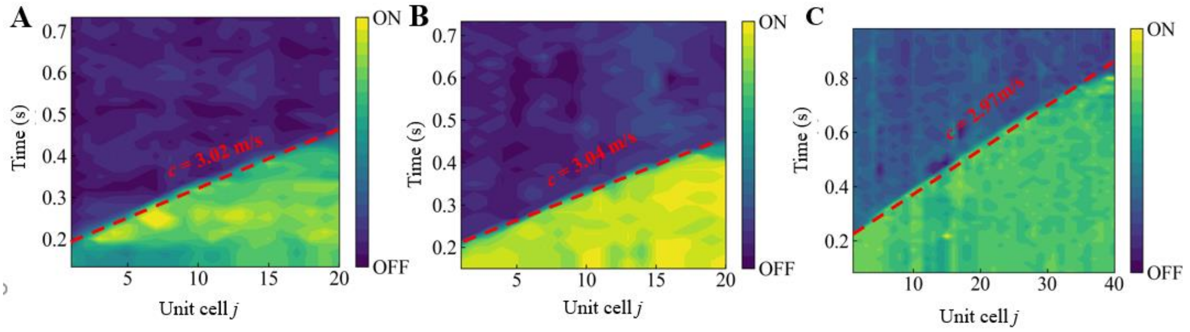

Fig. S18 The non-friction experimental evolution rule of medium transition wave velocity (θ=0°): (A) 20-units chain with swapped front, **(B)** rear halves and **(C)** 40 units.

Text S4 Phase transitions perception via electromagnetic resonance

To investigate the effect of frequency differences on the classification accuracy, LC resonance differential equations with internal resistance are established and solved. This allows quick adjustment of resonant frequencies of the units, so that spectral datasets with different frequency differences can be constructed to test the robustness of the neural network. The five -unit resonant circuits are connected in series, as shown in Fig.S22.

According to Kirchhoff's current law and Ohm's law, a system of differential equations can be constructed.

$$\dot{\mathbf{I}} \cdot \mathbf{L} = \mathbf{U} \tag{24}$$

$$\mathbf{C}\dot{\mathbf{V}} = \mathbf{I} \tag{25}$$

$$\mathbf{C} = \begin{bmatrix} C_1 + C_2 & -C_2 & 0 & 0 & 0 \\ -C_2 & C_2 + C_3 & -C_3 & 0 & 0 \\ 0 & -C_3 & C_3 + C_4 & -C_4 & 0 \\ 0 & 0 & -C_4 & C_4 + C_5 & -C_5 \\ 0 & 0 & 0 & -C_5 & C_5 \end{bmatrix} \tag{26}$$

$$\mathbf{V} = [V_2 \quad V_3 \quad V_4 \quad V_5 \quad V_6]^{\mathrm{T}} \tag{27}$$

$$\mathbf{I} = [I_{L1} - I_{L2} \quad I_{L2} - I_{L3} \quad I_{L3} - I_{L4} \quad I_{L4} - I_{L5} \quad I_{L5}]^{\mathrm{T}} \tag{28}$$

$$\mathbf{L} = [L_1 \quad L_2 \quad L_3 \quad L_4 \quad L_5]^{\mathrm{T}} \tag{29}$$

$$\mathbf{U} = \begin{bmatrix} -V_2 \\ V_2 - V_3 \\ V_3 - V_4 \\ V_4 - V_5 \\ V_5 - V_6 \end{bmatrix} - \mathbf{R} \cdot \mathbf{I} \tag{30}$$

Where **I** is the column vector of currents flowing through L_1 to L_5, and **V** is the column vector of voltages at nodes N_2 to N_6. The values of each variable are as shown in Table S2.

Due to the perturbation of the j-th inductance value by the state of the ($j-1$)-th unit, its value is determined in Table S3:

We use the SciPy 1.0 package established by Oliphant, T.E. et al.(*61*) in python to solve this equation. Fig.S23 shows that the numerical model can accurately simulate spectral changes during unit state transitions. By varying the inductance, normally distributed random perturbations with a prescribed coefficient of variation (C_v) are introduced into the resonance frequencies.

To investigate the effect of frequency spacing and perturbations on robustness, the unit resonance frequencies are first normalized:

$$\mathbf{L}_{\mathrm{ON},i} = 2.6 \qquad i = 1,2,3,...,5 \tag{29}$$

$$\mathbf{L}_{\mathrm{OFF},i} = 7.0 \qquad i = 1,2,3,...,5 \tag{30}$$

$$\mathbf{L}_{\mathrm{OFF}^*,i} = 3.7 \qquad i = 1,2,3,...,5 \tag{31}$$

The Coefficient of Variation is used to evaluate this variability of the classifier. By using different resonant capacitances C, datasets are generated by adjusting the resonance peak frequency difference δ_f between units. Where δ_f is defined as the ratio of the frequency difference between adjacent units to their resonance frequency

$$\delta_f = \frac{f_{j+1} - f_j}{f_{j+1}} \tag{32}$$

As shown in Fig. S24A–C, the spacing between resonance frequencies increases with increasing δ_f. Fig.S24 C, D and E show the distribution of the dataset is cluttered and dispersed with the increase of Coefficient of Variation. The neural network is trained using the above dataset, and its accuracy is calculated as shown in Fig.S24F. It can be observed that smaller coefficient of variation lead to higher accuracy. When the frequency difference is below 0.05, the identification accuracy remains largely unchanged; however, it drops sharply when the difference exceeds 0.05. The machine learning can achieve greater than 95% accuracy when the *Cv* of the system resonant frequency is less than 0.048 and the resonance peak frequency difference δ_f is greater than 0.05.

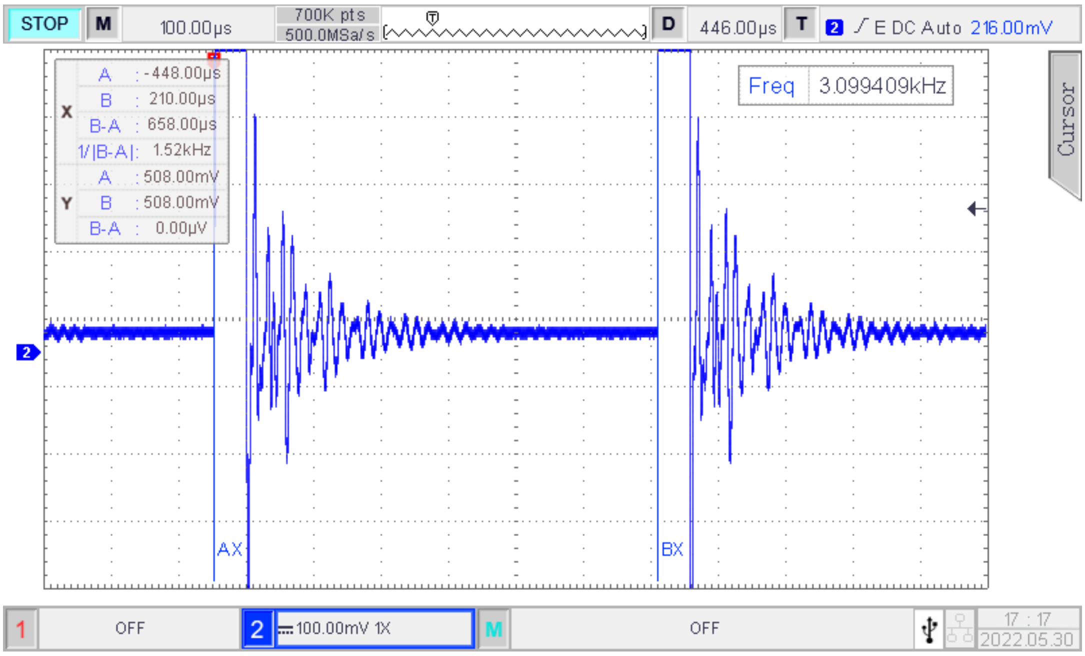

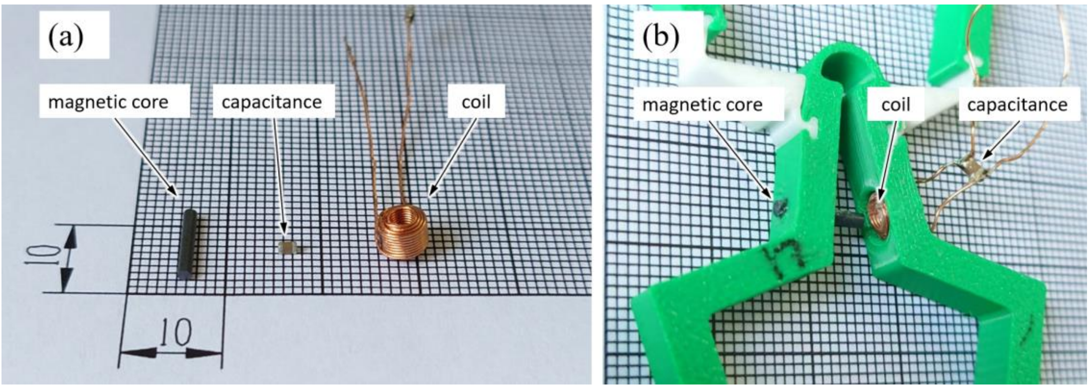

Fig. S19 Components used to artificial mechanoreceptors: **(A)** Independent magnetic core capacitor and coil; **(B)** Magnetic cores, capacitors and coils assembled into metamaterials.

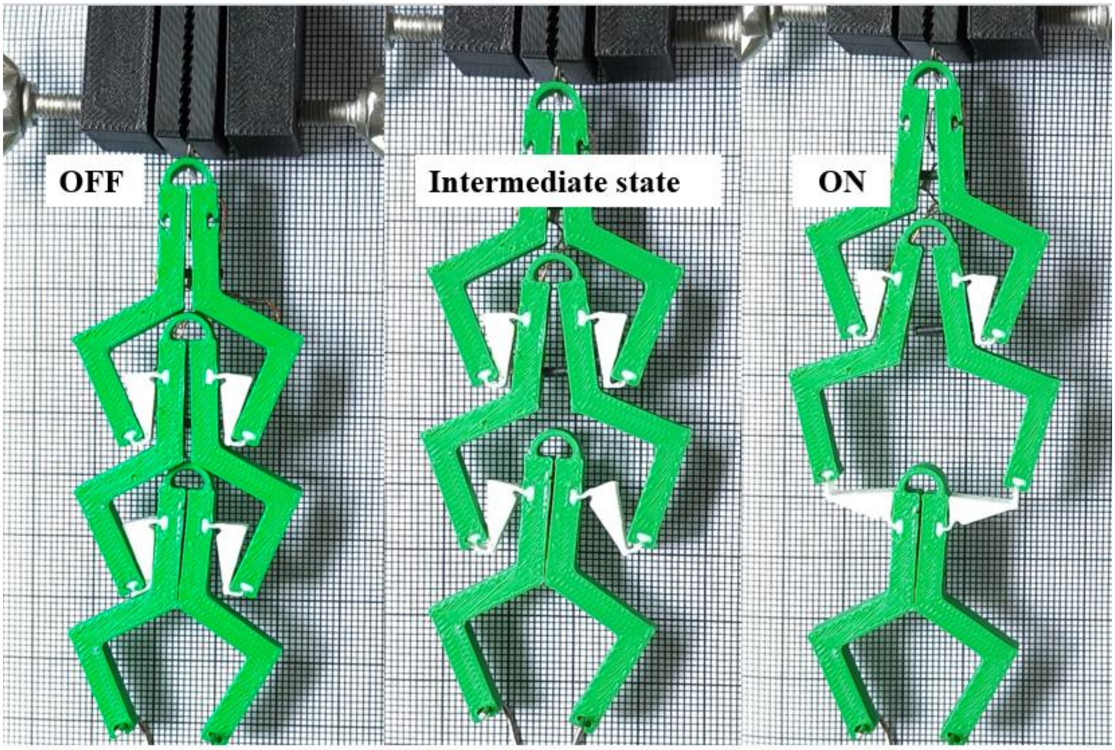

Fig. S20 Artificial mechanoreceptors in open, intermediate, and close state.

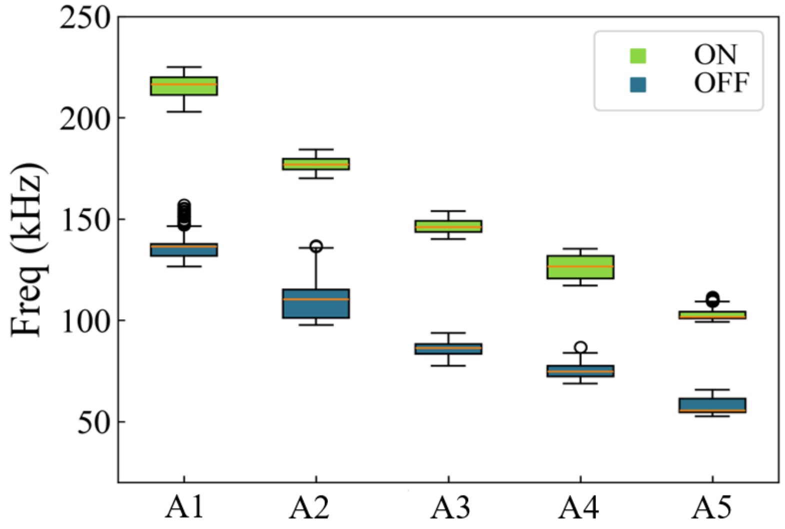

Fig. S21 The Box plot of the resonant frequency distribution of five units under ON and OFF state.

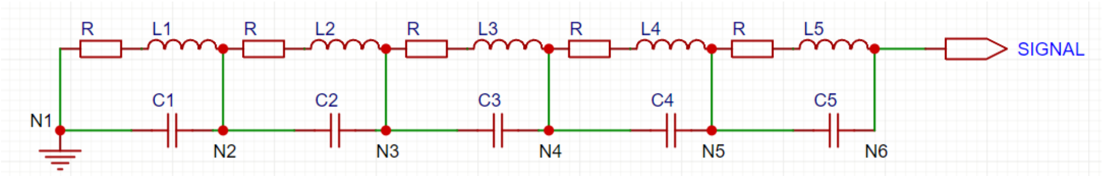

Fig. S22 The resonant circuit diagram with internal resistance.

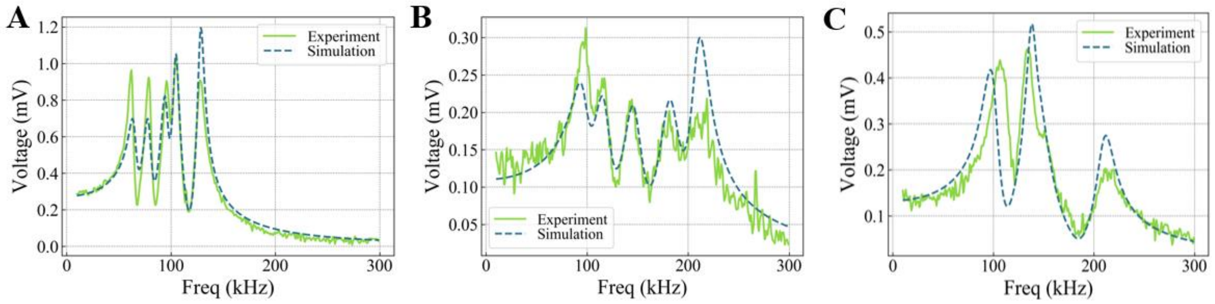

Fig. S23 Theoretical simulation and experimental resonance spectrum of five units:**(A)** the chain is OFF state; **(B)** the chain is ON state; **(C)** The chain is ON-OFF-ON-OFF-ON state.

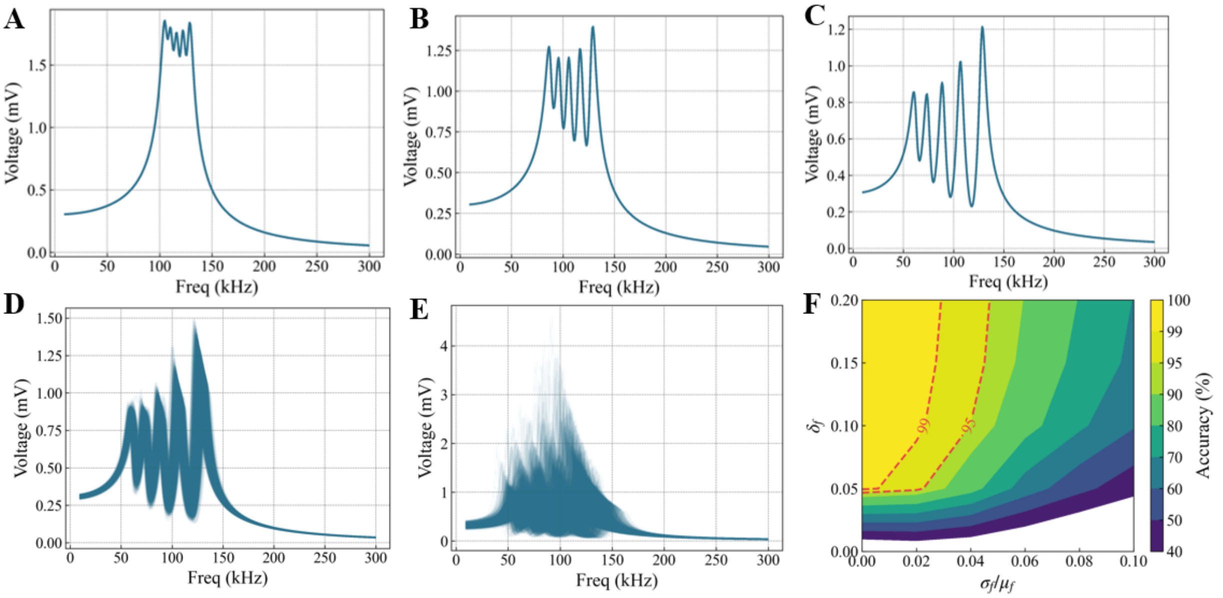

Fig. S24 Partial samples in dataset:(A) δ_f=0.05,σ_f / μ_f =0; **(B)** δ_f=0.1, σ_f / μ_f =0; **(C)** δ_f=0.2, σ_f / μ_f =0; **(D)** δ_f=0.2, σ_f / μ_f =0.02;**(E)** δ_f=0.2, σ_f / μ_f =0.1 **(F)** The effect of δ_f and σ_f / μ_f on recognition accuracy.

Table S1 The mean μ_f, minimum resonant frequency $f_{\min}$, minimum resonant frequency $f_{\max}$ and Coefficient of variation σ_f/μ_f of the mechanoreceptor array.

	μ_f	$f_{\min}$	$f_{\max}$	σ_f/μ_f
A1-ON	215.236	202.935	225.088	0.029
A1-OFF	135.919	126.587	157.047	0.038
A2-ON	177.104	170.102	184.343	0.019
A2-OFF	109.508	97.710	136.873	0.072
A3-ON	146.455	140.037	153.883	0.025
A3-OFF	85.609	77.535	93.754	0.042
A4-ON	126.332	117.093	135.290	0.046
A4-OFF	75.298	68.832	86.633	0.043
A5-ON	103.280	99.292	111.555	0.035
A5-OFF	57.106	52.613	65.667	0.066

Table S2 The parameters of resonant circuit simulation.

Variable	Value	Unit
$\mathbf{V}\|_{t=0}$	$[1.8, 3.6, 5.4, 7.2, 9.0]^T$	mV
$\mathbf{I}\|_{t=0}$	$[6.0, 6.0, 6.0, 6.0, 6.0]^T$	mA
R	$[0.3, 0.3, 0.3, 0.3, 0.3]^T$	ohms
$\mathbf{C}$	$[220, 330, 470, 680, 1000]^T$	nF
$\mathbf{L_{ON}}$	$[2.6, 2.3, 2.5, 2.7, 2.7]^T$	μH
$\mathbf{L_{OFF}}$	$[7.0, 7.0, 6.0, 6.0, 6.0]^T$	μH
$\mathbf{L_{NEAR}}$	$[3.8, 3.8, 3.7, 3.7, 3.7]^T$	μH

Table S3 Parameter table for the *j*-th and (*j*-1)-th unit.

	j-th ON	*j*-th OFF
(*j*-1)-th ON	$L_{ON,j}$	$L_{OFF*,j}$
(*j*-1)-th OFF	$L_{ON,j}$	$L_{OFF,j}$

Text S5 Information encoding and computation by phase transition perception

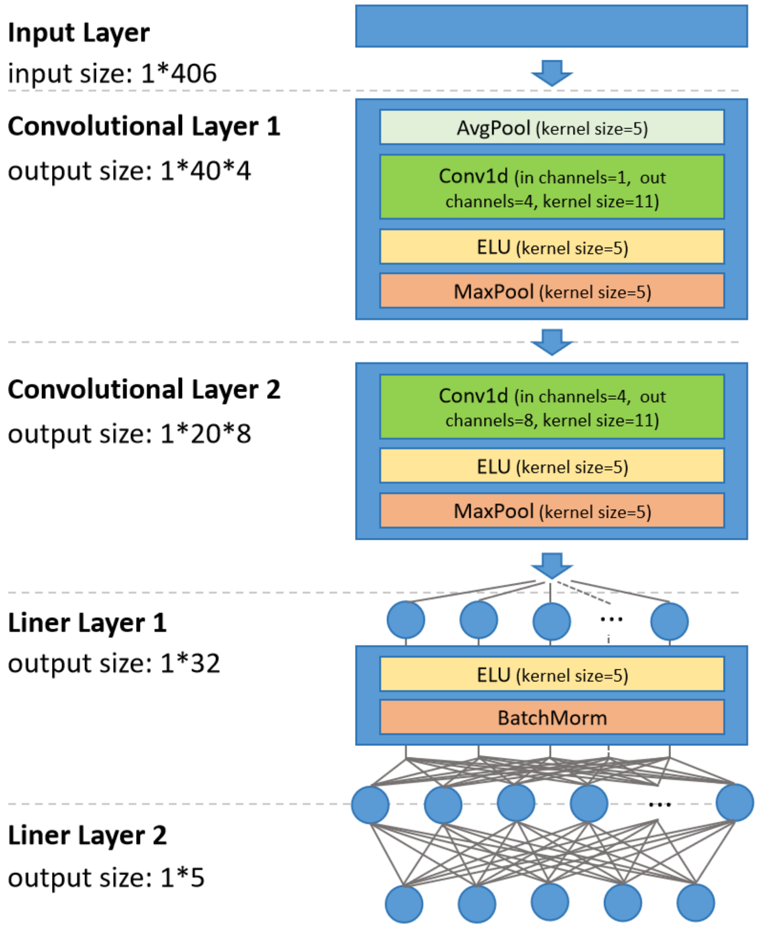

Fig. S25 Structure diagram of convolutional neural network.

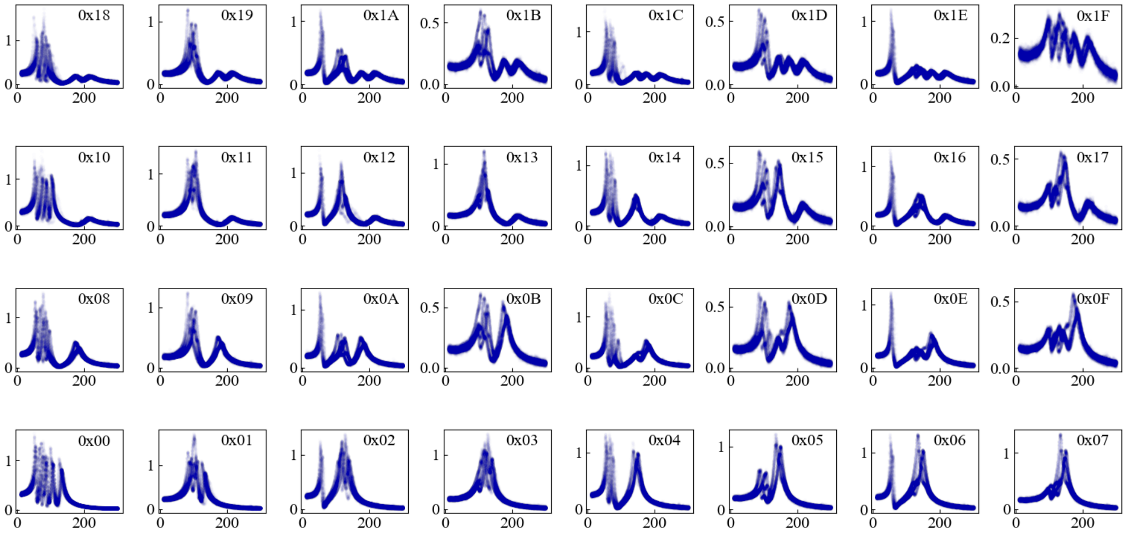

Fig. S26 Partial dataset of mechanoreceptor array. The abscissa bit frequency of each sub graph, in kHz, and the ordinate is voltage, in MV. The hexadecimal number in the upper right corner indicates the status of the chain, with on as 1 and off as 1. For example, the hexadecimal value “0x05” corresponds to the binary sequence “00101”, meaning the unit states are OFF, OFF, ON, OFF, and ON, respectively.

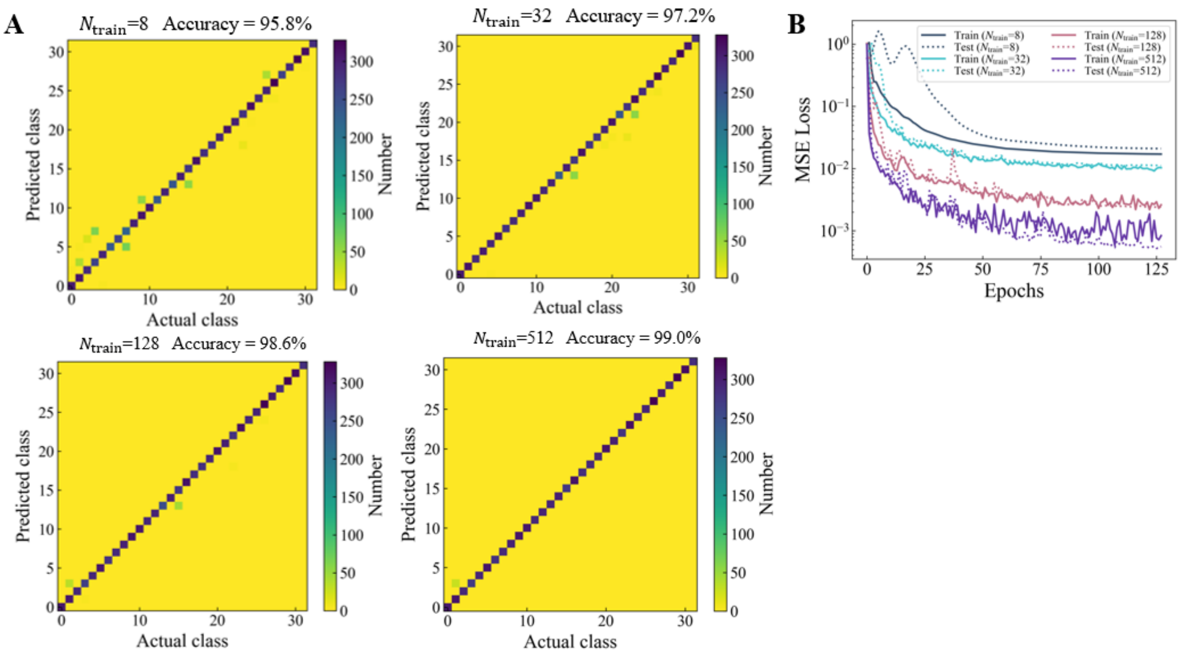

Fig. S27 Recognition accuracy and MSE loss of machine learning models with different data volumes $N_{\mathbf{train}}$. **(A)** Recognition accuracy with different data volume; **(B)** Loss function variation with different data volume.

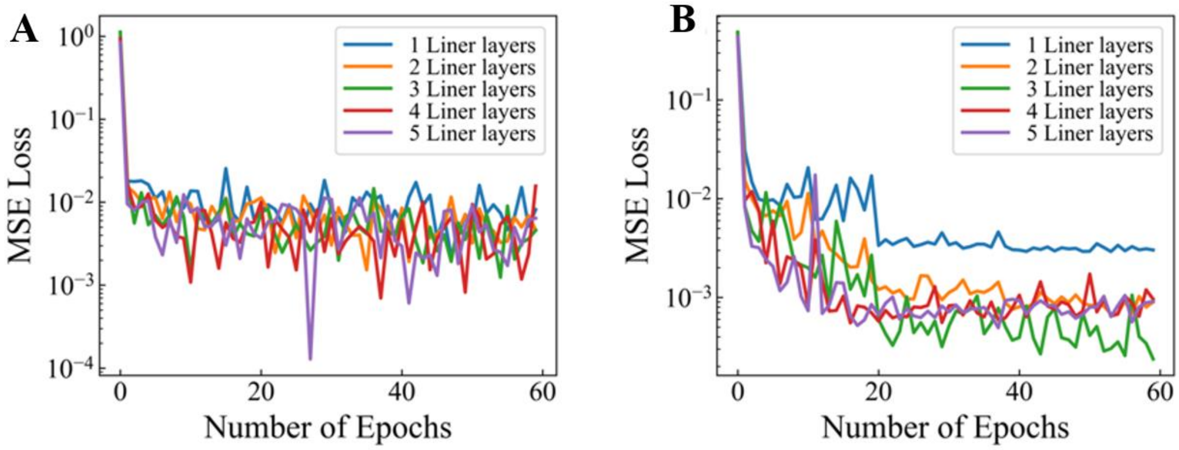

Fig. S28 MSE Loss function values during training with different linear layers: (A) training set; **(B)** testing test.

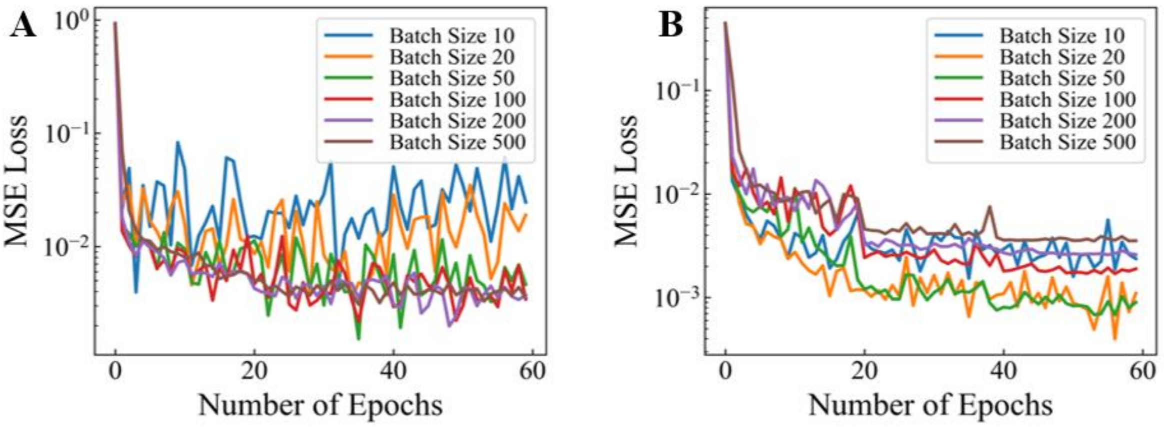

Fig. S29 MSE Loss function values during training with different batch sizes: (A) training set; **(B)** testing test.

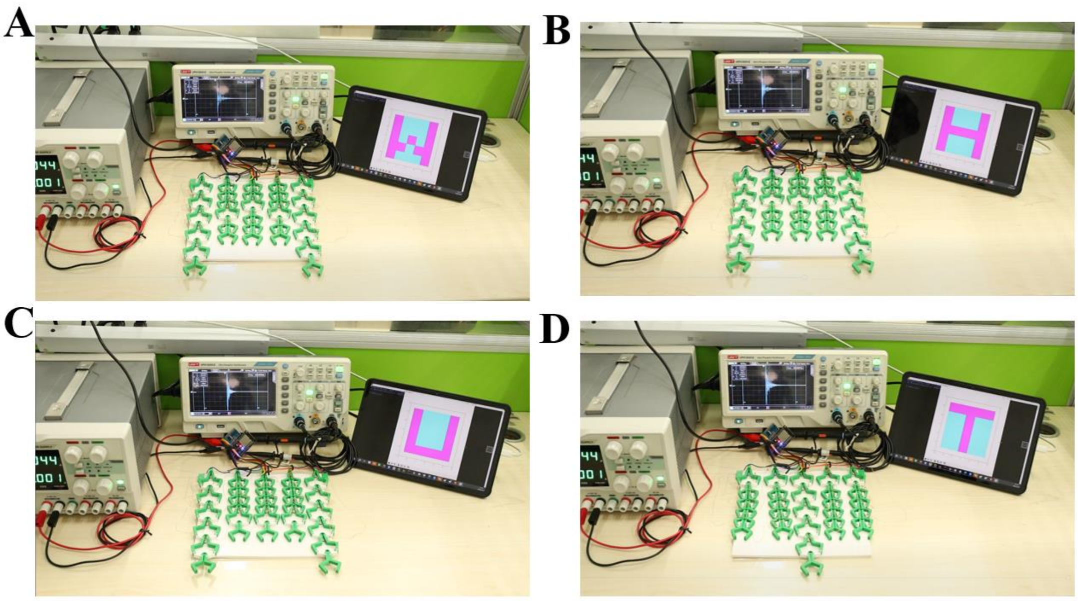

Fig. S30 Various states and their corresponding encoding patterns: (A) W; **(B)** H; **(C)** U; **(D)** T.

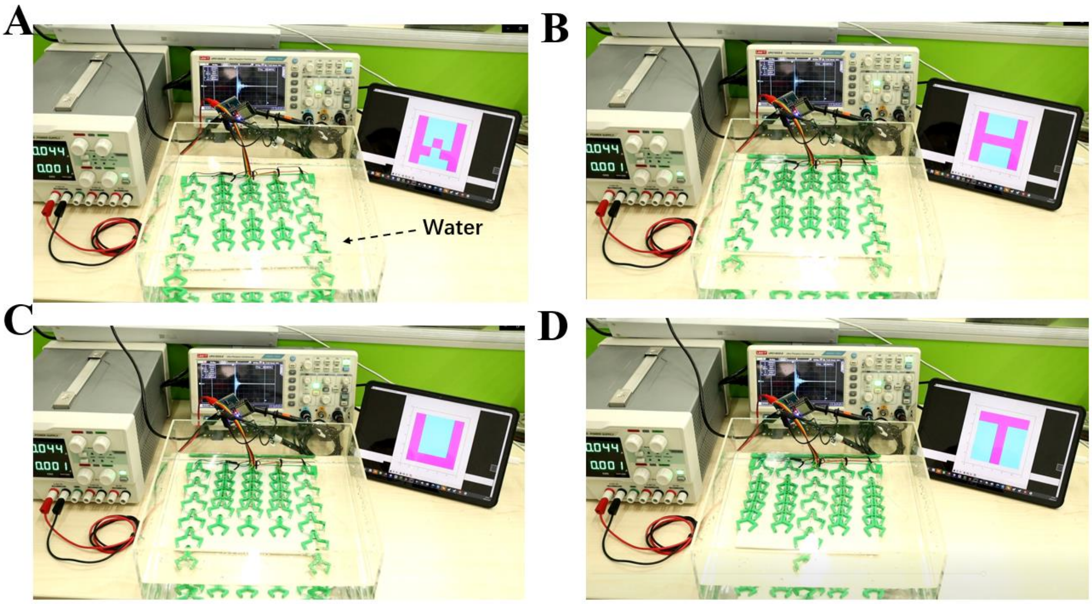

Figure S31 Various states and their corresponding encoding patterns in water environment: (A) W; **(B)** H; **(C)** U; **(D)** T.

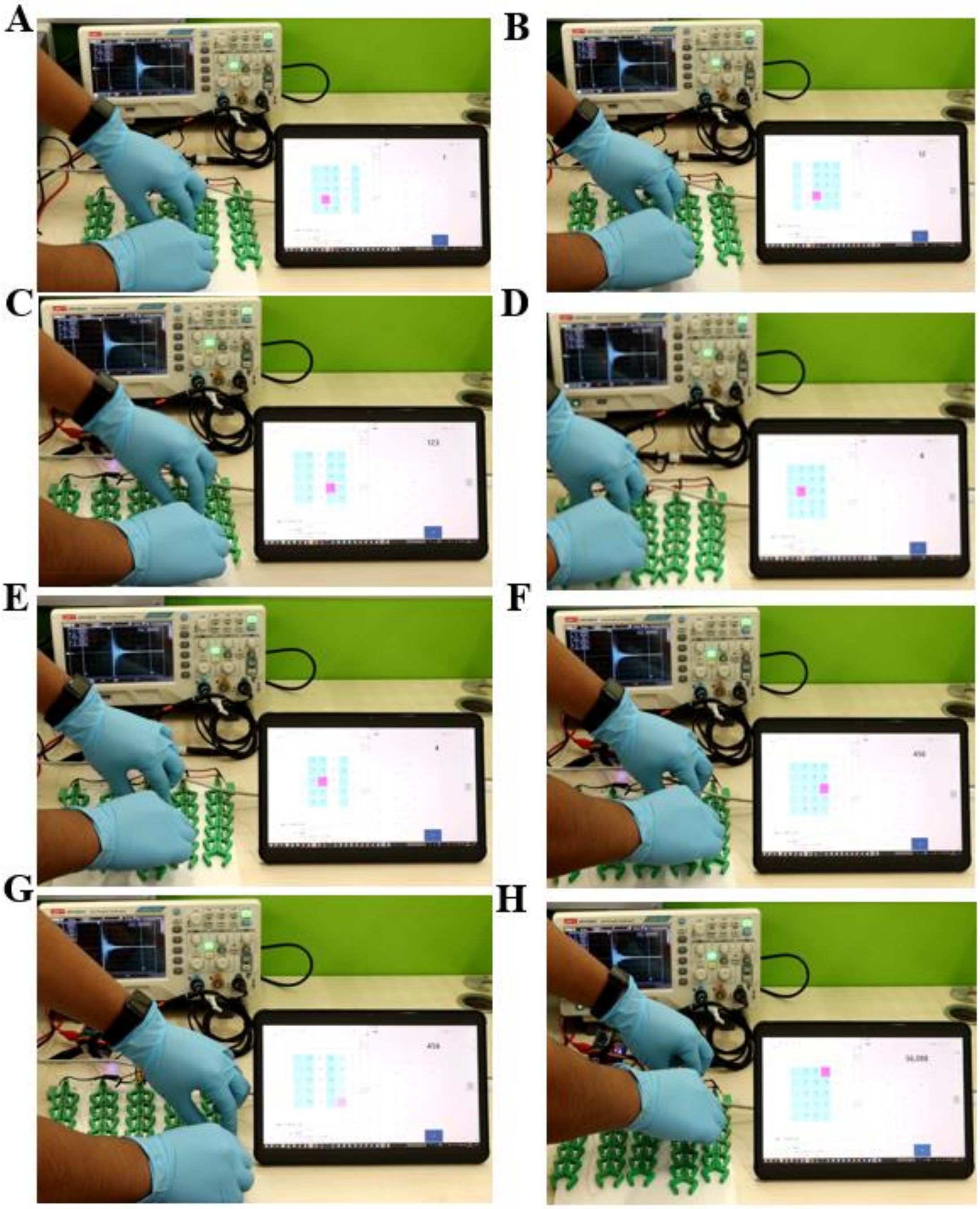

Figure S32 Demonstration of real-time information computation using the mechanoreceptor array as a keypad. (A) 1; **(B)** 2; **(C)** 3; **(D)**×; **(E)** 4; **(F)** 5; **(G)** 6; **(H)** =.

Text S6 Solitary wave-driven hydraulic propulsion in soft, self-sensing robots

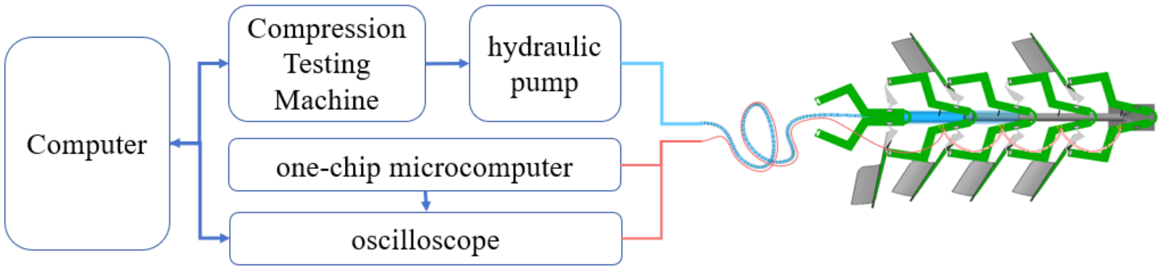

Figure S33 Working principle of a robot driven by electromagnetic resonance and solitons.

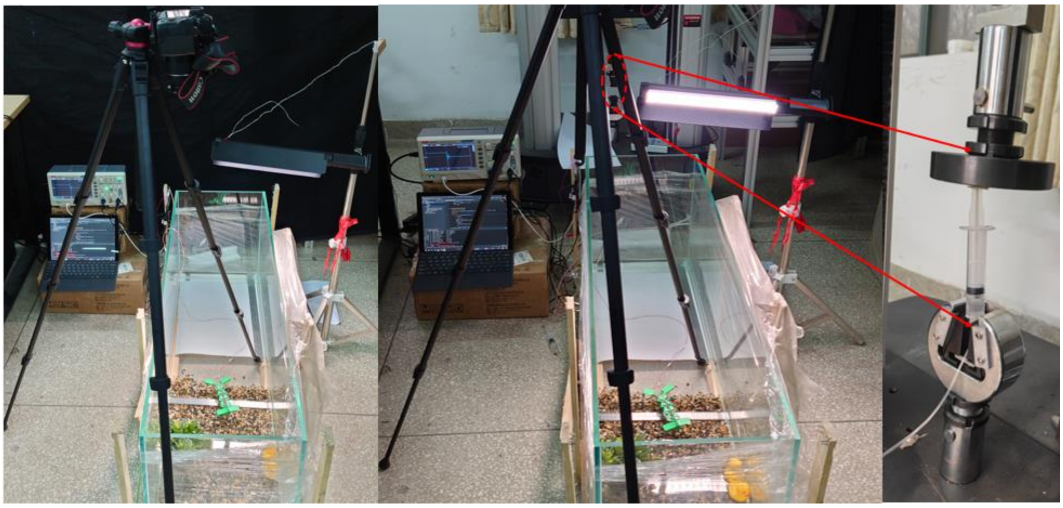

Figure S34 Schematic of the experimental setup for robotic motion measurement.

Table S4 Comparison of soft robots with different actuation, sensing, and control strategies.

	**Actuation method**	**Self-sensing method**	**Control mechanism**	**Operating environment**	**BL/cycle**
**This work**	Hydraulic pressure	electromagnetic resonance	Topological soliton	Locomotion on aquatic surfaces	~0.33
**Ref.1(*62*)**	Pneumatic pressure	No	Kirigami instabilities	Locomotion on flat lands	~0.15
**Ref.2(*63*)**	Pneumatic pressure	No	Vector soliton	Locomotion on flat lands	~0.45
**Ref.3(*64*)**	Electricity-driven	electromagnetic resonance	No	Locomotion on aquatic surfaces	~0.17
**Ref.4(*65*)**	Pneumatic pressure	No	No	Locomotion on flat lands	~0.02
**Ref.5(*66*)**	Pneumatic pressure	No	Structural stiffness change and buckling deformation	Locomotion on aquatic and flat lands	~0.38
**Ref.6(*67*)**	Electro-driven	No	Temperature-controlled deformation of LCE	Locomotion on flat and aquatic surfaces	~0.19

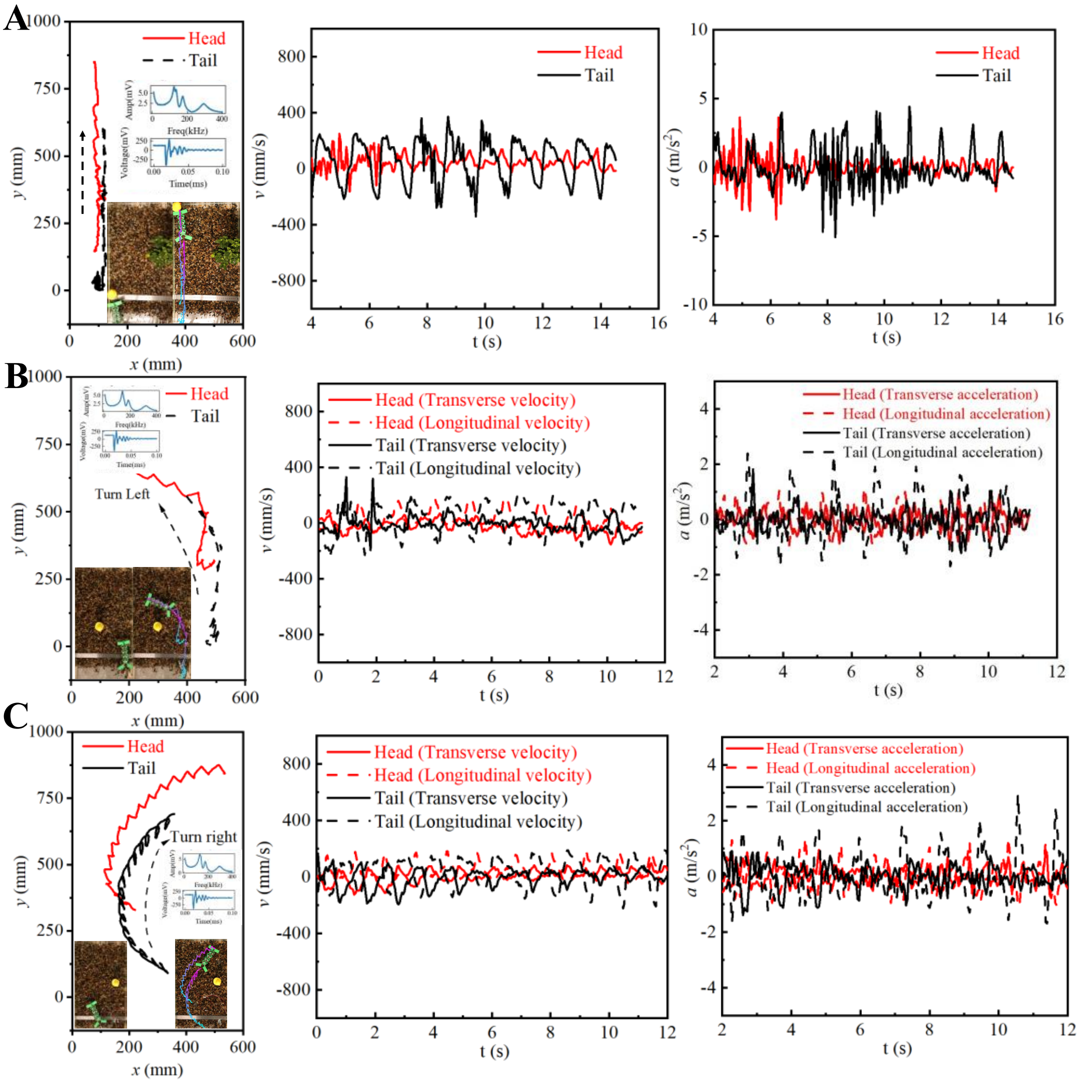

Figure S35 Kinematic responses of the robot head and tail during different locomotion scenarios. (A) transport cargo; **(B)** turn left; **(C)** turn right.

Supplementary Movie Captions:

Movie S1 Comparison of mechanical response between rigid hinges and soft ligaments.

Movie S2 Ethanol (stained red) being guided to the left by the Crassula muscosa, but not to the right.

Movie S3 Metamaterials consisting of fourteen identical unit cells exhibits non-reciprocity in dynamic model from right to left.

Movie S4 Metamaterials consisting of fourteen identical unit cells exhibits non-reciprocity in dynamic model from left to right.

Movie S5 Experimental and theoretical results of propagation with the transition wave consisting of 40 cells.

Movie S6 Experimental results of propagation with the tunable transition wave velocity by adjusting the geometric parameters.

Movie S7 Various states and their corresponding encoding patterns using the mechanoreceptor array

Movie S8 Various states and their corresponding encoding patterns in water environment using the mechanoreceptor array.

Movie S9 Real-time information computation using the mechanoreceptor array as a keypad.

Movie S10 Trajectories of robotic locomotion on the water: Straight ahead.

Movie S11 Trajectories of robotic locomotion on the water: transport cargo.

Movie S12 Trajectories of robotic locomotion on the water: turn left.

Movie S13 Trajectories of robotic locomotion on the water: turn right.

Supplementary code caption:

Code S1 The code included dynamicdata, staticdata and electromagnetic resonance.
